# SegClarity: An Attribution-Based XAI Workflow for Evaluating Historical Document Layout Models

**DOI:** 10.3390/jimaging11120424

**Published:** 2025-11-28

**Authors:** Iheb Brini, Najoua Rahal, Maroua Mehri, Rolf Ingold, Najoua Essoukri Ben Amara

**Affiliations:** 1 Ecole Nationale d’Ingénieurs de Sousse, Laboratory of Advanced Technology and Intelligent Systems (LATIS), Université de Sousse, Sousse 4054, Tunisia; maroua.mehri@eniso.u-sousse.tn (M.M.); najoua.benamara@eniso.rnu.tn (N.E.B.A.); 2 DIVA Group, University of Fribourg, 1700 Fribourg, Switzerland; najoua.rahal@unifr.ch (N.R.); rolf.ingold@unifr.ch (R.I.)

**Keywords:** deep neural networks, document layout analysis, semantic segmentation, explainable artificial intelligence, attribution maps, evaluation metrics

## Abstract

In recent years, deep learning networks have demonstrated remarkable progress in the semantic segmentation of historical documents. Nonetheless, their limited explainability remains a critical concern, as these models frequently operate as black boxes, thereby constraining confidence in the trustworthiness of their outputs. To enhance transparency and reliability in their deployment, increasing attention has been directed toward explainable artificial intelligence (XAI) techniques. These techniques typically produce fine-grained attribution maps in the form of heatmaps, illustrating feature contributions from different blocks and layers within a deep neural network (DNN). However, such maps often closely resemble the segmentation outputs themselves, and there is currently no consensus regarding appropriate explainability metrics for semantic segmentation. To overcome these challenges, we present SegClarity, a novel workflow designed to integrate explainability into the analysis of historical documents. The workflow combines visual and quantitative evaluations specifically tailored to segmentation-based applications. Furthermore, we introduce the Attribution Concordance Score (ACS), a new explainability metric that provides quantitative insights into the consistency and reliability of attribution maps. To evaluate the effectiveness of our approach, we conducted extensive qualitative and quantitative experiments using two datasets of historical document images, two U-Net model variants, and four attribution-based XAI methods. A qualitative assessment involved four XAI methods across multiple U-Net layers, including comparisons at the input level with state-of-the-art perturbation methods RISE and MiSuRe. Quantitatively, five XAI evaluation metrics were employed to benchmark these approaches comprehensively. Beyond historical document analysis, we further validated the workflow’s generalization by demonstrating its transferability to the Cityscapes dataset, a challenging benchmark for urban scene segmentation. The results demonstrate that the proposed workflow substantially improves the interpretability and reliability of deep learning models applied to the semantic segmentation of historical documents. To enhance reproducibility, we have released SegClarity’s source code along with interactive examples of the proposed workflow.

## 1. Introduction

Historical documents preserved in museums, libraries, and archives represent an essential part of the cultural heritage of human history and civilization, containing valuable information that can provide deep insights into the past [1]. However, these documents are highly vulnerable to degradation over time. Consequently, the effective protection, preservation, and valorization of such materials has become an urgent objective. A widely adopted solution is to convert them into digital form. In recent years, the digitization of historical documents has emerged as a paramount task. As a result, millions of digitized documents are now stored on servers, offering rapid and remote access. Beyond this significant achievement in terms of accessibility and preservation, the next major step and real promise of digitization lies in making the content of these vast collections of stored documents exploitable. Ongoing efforts aim to provide researchers with the tools and opportunities to apply computational techniques that advance historical document image processing across numerous tasks. This momentum is particularly evident in the area of layout analysis, which has witnessed a remarkable increase in research initiatives over the past few decades [2].

**Figure 1:**
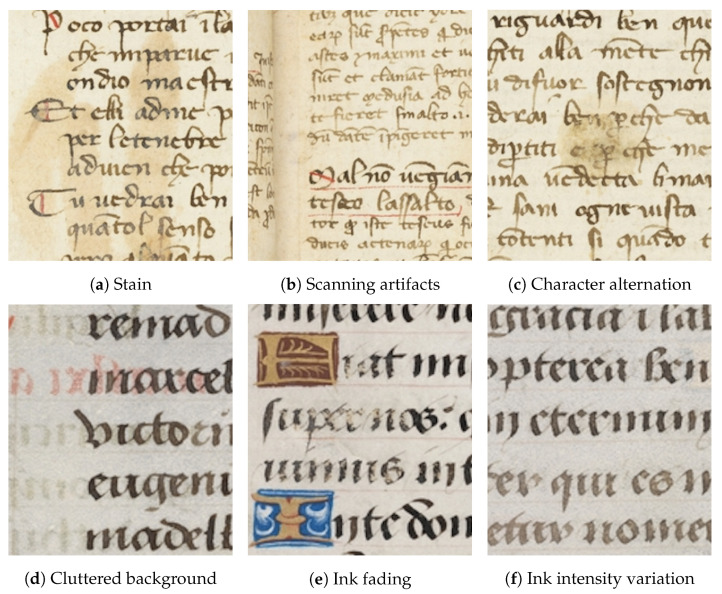
Zoomed-in regions of HDIs exhibiting various types of degradation. Examples are drawn from the CB55 [19] and UTP-110 [20] datasets.

Layout analysis is a fundamental component of document image processing and a prerequisite for text recognition. It enables the segmentation of a document into semantically homogeneous units such as background, text blocks, tables, and other structural elements [3]. The main challenges of layout analysis in Historical Document Images (HDIs) stem from the heterogeneity and complexity of layouts, as well as from various degradations. As illustrated in Figure 1, HDIs suffer from various forms of degradations such as stain, scanning artifacts, character alteration due to stain or damage, cluttered background, ink fading, and ink intensity variation.

Recent advances in document image processing and multimedia security have also demonstrated the effectiveness of hybrid-domain approaches that integrate multiple transform techniques to improve robustness, stability, and interpretability. Shubuh et al. [4] proposed a hybrid watermarking scheme that distributes watermark data across multiple transform blocks to resist geometric and noise attacks while maintaining high perceptual quality. Similarly, Kusuma and Panggabean [5] combined the Discrete Wavelet Transform (DWT), Hessenberg Decomposition (HD), and Singular Value Decomposition (SVD) to achieve enhanced numerical stability and imperceptibility under filtering, noise, and compression conditions. In a complementary review, Amrullah and Aminuddin [6] examined fragile and semi-fragile watermarking methods for tamper detection and restoration, emphasizing the need for adaptive algorithms that maintain both visual fidelity and robustness. Collectively, these studies underline how hybrid-domain fusion and intelligent restoration strategies can strengthen the reliability and interpretability of imaging systems. The introduction of deep neural networks (DNNs) has enabled recent research to substantially solve many challenges of HDIs by achieving near-perfect accuracy in different layout analysis tasks, particularly through pixel-wise semantic segmentation. They generally focus on page segmentation [7,8] as well as text line detection and classification [9,10,11,12]. However, these models are commonly considered as “black boxes”, which pose significant challenges in rendering their predictions interpretable to humans. Not even engineers or data scientists are able to clearly interpret what happens inside these models or how their results are generated. Explainable Artificial Intelligence (XAI) has emerged as a response to these challenges, aiming to enhance the transparency and interpretability of DNNs [13,14]. In the context of HDIs analysis, the need for XAI has become particularly pressing, as it provides clear and concise explanations of model predictions.

The XAI approaches can broadly be categorized into attribution-based, perturbation-based, attention-based, and transformer-based approaches [14]. In this paper, we focus on attribution-based approaches, also referred to as visualization approaches [15]. The attribution-based approaches generally aim to highlight the most relevant features or attributes in the input that influence the model’s decision, typically through various forms of visual representation. The most common approaches are the Gradient-based methods that leverage gradients to identify the most influential input features contributing to a model’s predictions. These methods analyze how small changes in input affect the model’s output, highlighting critical regions in images for interpretability. The most widely used techniques are Layerwise Relevance Propagation (LRP) [16], DeepLift [17], and GradCAM [18].

In this work, we present **SegClarity** at https://github.com/iheb-brini/SegClarity (access on 8 November 2025), a continuation of our previous framework **DocSegExp** [21]. SegClarity expands the methodological scope and analytical depth of explainable segmentation models by integrating additional XAI methods, refined evaluation metrics, and broader experimental validation. The key distinctions between the two works are summarized below:

- **Broader XAI Method Integration:** SegClarity extends beyond the limited set of attribution-based methods used in DocSegExp by incorporating four XAI techniques: *rad-CAM*, *DeepLIFT*, *LRP*, and *Gradient × Input*.
- **Compatibility with Perturbation-Based XAI:** SegClarity is fully compatible with perturbation-based methods such as *RISE* and *MiSuRe*, revealing that document layout analysis, unlike other segmentation domains, supports only a subset of XAI techniques that can effectively capture its complex structural characteristics (see Section 5).
- **Layer-Wise Attribution Analysis:** Unlike DocSegExp, which generated attributions solely from the final layer, SegClarity performs multilayer attribution analysis across five U-Net decoder layers, enabling a deeper understanding of model behavior throughout the feature hierarchy.
- **Expanded Evaluation Metrics:** While DocSegExp relied primarily on *ADCC* and *wADCC*, SegClarity introduces a more comprehensive evaluation suite, including *Infidelity* [22], *Sensitivity* [22], *content heatmap (CH)* [23], *pointing game (PG)* [24], and our novel *Attribution Concordance Score (ACS)* for assessing both robustness and interpretability.
- **Dual Evaluation Framework:** SegClarity introduces a combined quantitative and qualitative evaluation strategy. Quantitative metrics measure faithfulness and stability, while qualitative visualizations enhanced through post-processing and normalization improve interpretability and human alignment.
- **Enhanced and Diverse Experimental Validation:** DocSegExp was evaluated only on a synthetic dataset, whereas SegClarity includes two real-world historical document datasets (*CB55* and *UTP-110*), offering a more diverse and robust validation.
- **Cross-Domain Generalization:** Finally, beyond document layout analysis, SegClarity demonstrates domain transferability by successfully adapting the workflow to urban scene segmentation using the *Cityscapes* dataset, confirming its generalizability across semantic segmentation domains.

In this paper, we investigate the explainability of DNNs in the analysis of HDIs, with a particular focus on richly decorated medieval manuscripts. These documents present specific challenges due to the presence of diverse ornaments and decorations that need to be distinguished. Additional difficulties include decorative scripts and ink bleed-through, as well as colorful and intricately decorated objects.

The main contributions of this work are summarized below:

1. We propose SegClarity, a comprehensive workflow dedicated to evaluating segmentation-based applications through advanced visualization and normalization tools and using a broad set of evaluation metrics. SegClarity contributes to having human-understandable DNNs specifically tailored for pixel-wise semantic segmentation of HDI.
2. We adapt and evaluate six state-of-the-art XAI methods and four metrics for HDI, enabling a rigorous assessment of model explanation quality.
3. We present a targeted perturbation technique that leverages semantic annotations to generate meaningful perturbations, specifically designed to enable the accurate evaluation of interpretability in pixel-wise prediction tasks with faithfulness-based metrics.
4. We introduce a metric, called the Attribution Concordance Score (ACS), designed to enhance the robustness of explainability assessments. This metric is tailored to evaluate the alignment of model attributions with the semantic and structural characteristics of HDI layouts. We demonstrate the effectiveness of our metric both quantitatively, by comparison with two state-of-the-art metrics, and qualitatively, through expert and non-expert human assessments across three different datasets.
5. We evaluate the domain transferability of the proposed workflow and validate its generalization by extending our experiments beyond HDI analysis to include Cityscapes, one of the most widely used and complex benchmarks in the state-of-the-art for urban scene segmentation.

The remainder of this paper is organized as follows. Section 2 is dedicated to a literature review of layout analysis, semantic segmentation, and explainablity. Section 3 introduces the proposed attribution-based XAI workflow, presents the adapted evaluation metrics, and details both the targeted perturbation technique and the proposed ACS metric. Section 4 describes the experimental corpora and protocol, analyzes the results, and concludes with a comparative study of XAI metrics. Section 5 extends our work by illustrating the use of SegClarity on a different application domain, namely, Urban Street View, with the Cityscapes dataset. Section 6 highlights key investigations, findings, and limitations. Finally, Section 7 presents our conclusion and further work.

## 2. Literature Review

### 2.1. Explainable Semantic Segmentation Models

The general context of our work is semantic segmentation, which is the process of grouping regions of an image into classes at the pixel level. It means that each pixel in an image is assigned to a class label. Recently, several research studies have been conducted to propose reliable semantic segmentation of HDIs. In this section, we present the most relevant works in this area. Then, an overview of XAI methods applied to semantic segmentation is provided.

Grüning et al. [9] proposed ARU-Net, a variant of the U-Net model [25], for baseline detection in historical documents. Boillet et al. [26] presented the Doc-UFCN model, inspired by the dhSegment model [8], for text line segmentation. Rahal et al. [12] proposed L-U-Net to address two sub-tasks of layout analysis of HDI: page segmentation and text line detection. They showed that a smaller network with fewer parameters is well suited for the semantic segmentation of HDI. Rahal et al. [11] addressed the text line detection and classification with transfer learning strategies when only a few annotated training data are available. Da et al. [27] introduced a novel model called the Vision Grid Transformer (VGT), specifically designed for document layout analysis. Binmakhashen et al. [28] presented an extensive review of various methods and approaches used in document layout analysis. They cover techniques for page segmentation, text zone detection, text line extraction, and character recognition.

The previously mentioned works highlight the significance of the results achieved by deep learning models in the field of semantic segmentation for historical document analysis. Nevertheless, a major limitation persists: the absence of mechanisms that allow humans to understand, interpret, and trust the outputs generated by these models. This shortcoming is mainly due to the fact that the integration of XAI in this field remains largely unexplored. In contrast, most existing applications of XAI have focused on image classification tasks. For an accessible introduction to explainable image classification, we refer the reader to [29], while a more comprehensive survey on the subject can be found in [14]. However, despite being a ubiquitous task, interpretability in semantic image segmentation has not received the same level of attention. It remains a particularly challenging area of research. While it can be regarded as an extension of the relatively more intuitive task of interpretable image classification, it requires accounting for the combined influence of individually classified pixels of interest [30]. In the following, we present the most relevant works on XAI applied to semantic image segmentation.

In [31], Seg-Grad-CAM was introduced as an extension of Grad-CAM [18]. It was among the most well-known explainability techniques for semantic image segmentation. The generated saliency maps were obtained through a weighted sum of selected feature maps. Its effectiveness was demonstrated on a U-Net model trained on the Cityscapes [32] dataset. Hasany et al. [33] proposed MiSuRe, a model-agnostic two-stage method for generating saliency maps in image segmentation. They demonstrated its applicability on three diverse datasets: an artificial dataset (Triangle Dataset) [34], a medical dataset (Synapse multiorgan CT) [35], and a natural dataset (COCO-2017) [36], using both convolutional and transformer-based architectures.

The previously mentioned works indicate that the main application domains of XAI for semantic image segmentation have been focused on medicine and industry, such as building detection, pedestrian environments, and common objects. To the best of our knowledge, we are the first to tackle the challenge of applying XAI to historical document images, a particularly complex and underexplored domain.

In this context, we proposed DocSegExp, the first framework to extend the XAI for semantic segmentation of HDIs [21]. We introduced an adaptation of the evaluation metric ADCC [37], originally developed for classification, and proposed wADCC (weighted ADCC), a modification of the original metric. The metric wADCC is computed by considering the weighted average of each target class ADCC and its pixel distribution. The three attribution-based XAI methods, GradCAM [18], Gradient × Input [38], and DeepLIFT [17], were also repurposed from the classification task to the semantic segmentation of HDIs. The experiments were conducted on SynDoc12K [39], a synthetic dataset comprising 12,000 annotated historical document images, using two architectures: FCN101 [40] and ResUNet [41]. The obtained results revealed that the predictions made with ResNet outperformed those of FCN, although the ADCC metric indicated the opposite. This discrepancy arose because ADCC does not handle target classes equitably. In contrast, by assigning a weight to each class in the wADCC metric, we ensured a more balanced evaluation, which subsequently reflected the superior performance of ResNet over FCN in the predictions.

In [42], we proposed DocXAI-Pruner, an explainable AI-driven approach for pruning semantic segmentation models HDIs. Traditional pruning techniques rely on gradients or filter magnitudes. In contrast, DocXAI-Pruner utilizes Grad-CAM attribution maps to assess the contribution of individual channels, identifying those that are consistently less relevant across foreground target classes, while excluding the background class. Experiments were carried out on two UNet variants: the standard UNet (∼31 M parameters) [25] and L-U-NET (∼17,000 parameters) [12]. Models were trained on synthetic HDIs and fine-tuned on the CB55, a subset of the DIVA-HisDB dataset [19]. Both structured and unstructured pruning strategies were tested, with fine-tuning configurations including full retraining, freezing pruned layers, and freezing individual channels. Results show that unstructured pruning combined with channel-freezing fine-tuning achieved the best trade-off, with parameter reductions up to 26% and improved or preserved performance. In particular, standard UNET benefited most from pruning, achieving notable accuracy gains despite reduced complexity, whereas L-U-NET showed limited improvement due to its already compact design.

### 2.2. XAI Method Categorization

**Figure 2:**
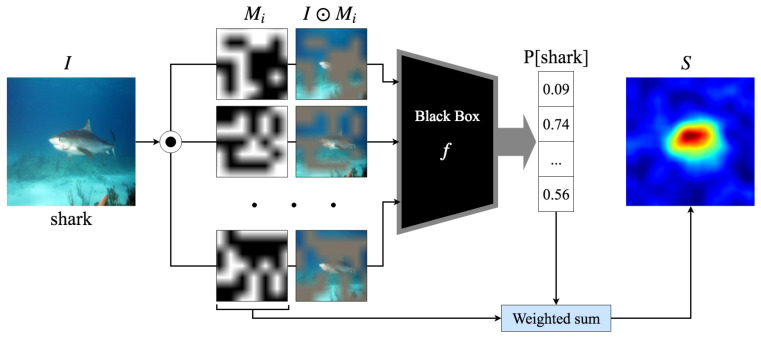
RISE explanation https://eclique.github.io/rep-imgs/RISE/rise-overview.png (accessed on 8 November 2025): The input image (I) is masked multiple times with random binary masks ($M_{i}$), generating occluded versions ($I⊙M_{i}$). Each masked image is fed through the black box model (f) to obtain class probabilities P[shark]. The final saliency map (S) is computed as a weighted sum of all masks, where weights correspond to their respective prediction scores.

The broader AI community has given rise to XAI, a burgeoning field focused on shedding light on the rationale behind model outputs. However, given the diverse nature of tasks, it is essential to customize XAI solutions for each specific domain, as different approaches are required to address the explainability challenge effectively. XAI methods have distinct characteristics, as outlined by the research community [13,43,44]. An *agnostic* method is designed to operate with any model architecture, irrespective of its complexity or internal structure. In contrast, most XAI methods are *model-specific*, meaning they are tailored for particular architectures, such as CNNs, recurrent neural networks (RNNs), or simple multilayer perceptrons (MLPs). Another key distinction lies in the scope of the explanations: some methods focus on explaining individual predictions, known as *local* explanations, while others aim to provide insights into the model’s overall behavior, referred to as *global* explanations. XAI methods can be categorized into three classes:

- **Gradient-based methods:** These are a class of XAI techniques that leverage gradients to identify the most influential input features contributing to a model’s predictions. These methods analyze how small changes in input affect the model’s output, highlighting critical regions in images for interpretability.
  One of the most widely used techniques is Grad-CAM, which generates a localization map by using gradients within a CNN to highlight critical regions for a target concept [18]. Grad-CAM uses the gradients of the target class score $y^{c}$ with respect to the feature map activations $A_{k}$ of a convolutional layer. The importance weight $\alpha_{k}^{c}$ for each feature map is computed as follows:
  (1) $$\alpha_{k}^{c}=\frac{1}{Z}\sum_{i}\sum_{j}\frac{\partial y^{c}}{\partial A_{k}^{ij}}$$
  where *c* is the target class, *k* is the filter index of a convolution layer, *i* and *j* denote the spatial coordinates of the feature map, and *Z* is the number of pixels in the feature map. The Grad-CAM heatmap $L^{c}$ is then obtained as follows:
  (2) $$L^{c}=\mathrm{ReLU}\left(\sum_{k}\alpha_{k}^{c}A_{k}\right)$$
  While Grad-CAM effectively identifies discriminative regions, it often produces coarse explanations due to its dependence on high-level feature maps. To overcome this limitation, *Guided Grad-CAM* [18] combines Grad-CAM with *Guided Backpropagation* [45], which preserves fine-grained details from lower convolutional layers. Guided Backpropagation modifies the backward ReLU operation by allowing only positive gradients to flow:
  (3) $$R^{l}=\left(R^{l+1}>0\right)⊙\left(x^{l}>0\right)⊙\frac{\partial x^{l+1}}{\partial x^{l}}$$
  where $R^{l}$ represents the relevance map at layer *l*, and $x^{l}$ denotes the activations.
  The final Guided Grad-CAM map is obtained by element-wise multiplying the high-resolution *Guided Backpropagation* map with the coarse *Grad-CAM* map:
  (4) $$L_{\mathrm{Guided}-\mathrm{GC}}^{c}=L_{\mathrm{Grad}-\mathrm{CAM}}^{c}⊙L_{\mathrm{GuidedBP}}^{c}$$
  G*I is a different gradient method that multiplies the gradient of the output with respect to the input by the input itself, producing a saliency map that identifies the most influential pixels on the outcome [38]. The attributions for each input pixel $x_{ij}$ in *x* are given by the following:
  (5) $$A_{ij}=x_{ij}\cdot \frac{\partial y}{\partial x_{ij}}$$
  where *i* and *j* denote the spatial coordinates of the input image.
  G*I provides an intuitive measure of how changes in the input directly affect the output prediction, highlighting influential regions in the image.
- **Perturbation-based methods:** These assess feature importance by systematically modifying input data and analyzing the model response to these alterations.
  Local interpretable model-agnostic explanations (LIME) perturbs input features and fits a locally interpretable model to approximate the behavior of complex models, highlighting the contribution of individual features [46].
  Similarly, Shapley additive explanations (SHAP) leverages Shapley values from cooperative game theory, systematically perturbing input features to quantify their influence on model predictions [47]. In the same vein, the RISE method [48] generates a large number of random binary masks that selectively occlude parts of the input image. Each mask’s contribution to the model prediction is measured, and the importance of each pixel is estimated as the weighted average of these random perturbations as described in Figure 2. Unlike LIME or SHAP, RISE is model-agnostic and gradient-free, making it applicable even to non-differentiable models.
  While RISE uses random perturbations to estimate pixel importance, MISURE [33] takes a more targeted approach by providing a dual explanation framework. MISURE systematically identifies both the minimal set of features sufficient for maintaining the current prediction (sufficiency) and the minimal modifications required to alter the prediction to a different class (necessity). Through an optimization-based formulation that balances explanation compactness and prediction confidence, MISURE delivers complementary perspectives on feature importance via sufficient and counterfactual explanations.
- **Decomposition-based methods:** These focus on breaking down a model decision-making process into interpretable components by redistributing relevance scores across input features. Unlike perturbation-based methods, which remove or modify input regions, decomposition-based techniques directly trace the flow of information through a neural network to determine how different parts of the input contribute to the output.
  A prominent example is LRP, which assigns important scores to input pixels by backpropagating relevance from the output layer to the input, ensuring that the total relevance is conserved across layers [16]. LRP redistributes relevance scores *R*, layer by layer, using the following propagation rule:
  (6) $$R_{p}=\sum_{q}\frac{z_{pq}}{\sum_{p}z_{pq}}R_{q}$$
  where $z_{pq}$ represents the contribution of neuron *p* to neuron *q*, and $R_{q}$ is the relevance of neuron *q* in the upper layer. The redistribution follows conservation principles, ensuring relevance is neither created nor lost.
  Another know method is DeepLIFT which calculates contribution scores by comparing neuron activations to a reference, enhancing traditional gradient methods by considering neuron reference activations [17]. DeepLIFT defines the contribution score *C* of an input neuron $x_{n}$ to an output neuron *y* as
  (7) $$C_{\Delta x_{n}}=\left(y-y_{\mathrm{ref}}\right)\cdot \frac{\Delta x_{n}}{\sum_{n}\Delta x_{n}}$$
  where $y_{\mathrm{ref}}$ is the reference activation, and $\Delta x_{n}$ is the difference between the actual and reference activation of neuron *n*.
  DeepLIFT ensures that small variations in the input, which do not significantly affect the output, receive small attributions.

In this paper, we evaluate six attribution methods, summarized in Table 1, covering diverse categories of explainability approaches. These include four gradient and decomposition methods and two perturbation-based techniques. We classify each method according to its ability to provide explanations at the input or intermediate layer levels. All six methods support input-layer explanations, while only the perturbation-based methods (*RISE* and *MiSuRe*) do not support layer-wise analysis.

For consistency, we adopt a unified notation for *Grad-CAM*: when applied to the input layer, we use its variant *Guided Grad-CAM*. However, for simplicity and readability, we collectively refer to both as *Grad-CAM* throughout the paper.

## 3. Proposed Attribution-Based XAI Workflow

In this work, we propose a comprehensive workflow that elucidates the layer contributions within a DNN. Our workflow utilizes the input image, the DNN parameters, the identified or target class, the chosen XAI technique, and a specific layer of interest within the network.

**Figure 3:**
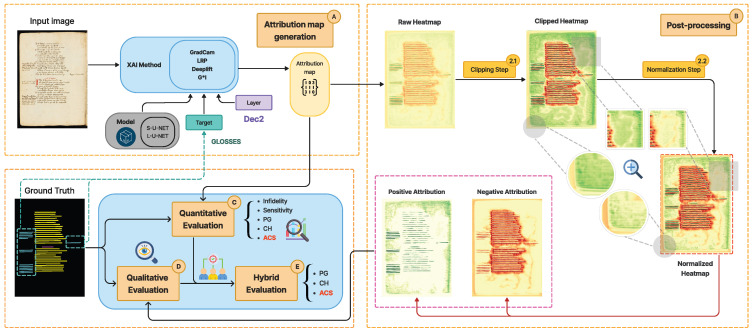
Overview of the proposed workflow. The workflow consists of five main steps: (1) Attribution map generation using a selected XAI method—*Grad-CAM*, *LRP*, *DeepLIFT*, *Gradient × Input*, or the perturbation-based methods *RISE* and *MiSuRe*—applied to a DNN with a chosen input image, target class, and selected input and intermediate layer in the network; (2) Post-processing to enhance the visual quality and interpretability of the generated attribution maps; (3) Qualitative evaluation through visual inspection and expert analysis; (4) Quantitative evaluation using objective metrics to assess consistency and reliability; and (5) Hybrid evaluation, combining both visual and metric-based assessments for comprehensive interpretability analysis.

This section follows the pipeline of the proposed workflow illustrated in Figure 3 and unfolds into five steps.

1. **Attribution map generation** has two complementary stages:
  (a) **Segmentation adaptation** reshapes dense segmentation outputs into a classification-like form (via the adapter in Algorithm A1);
  (b) **Attribution computation** using with four different XAI methods to generate layer-wise maps.
2. **Post-processing** improves readability through a clipping step to suppress outliers and a normalization step that separates positive/negative evidence.
3. **Quantitative evaluation** measures explanation quality using Infidelity with targeted perturbations, Sensitivity_max, content heatmap (CH), pointing game (PG), and our new novel metric, called the Attribution Concordance Score (ACS).
4. **Qualitative evaluation** visually inspects positive/negative and blended overlays on the input images to contextualize the maps.
5. **Hybrid evaluation** integrates the two evaluation phases into a hybrid evaluation, by leveraging human assessments of the generated heatmaps to examine how well the evaluation metrics align with human interpretation.

### 3.1. Attribution Map Generation

Attribution maps serve as heatmaps conveying XAI insights to interpret model decisions. As most attribution methods were initially designed for classification models that output a single score vector per image, their application to semantic segmentation requires specific adaptations. To address this challenge, this phase is divided into two complementary stages: segmentation adaptation and attribution computation, which are described below (Further theoretical details are provided in Appendix A.1.1 and Appendix A.1.2):

- **Segmentation adaptation:** We reshape the dense outputs of segmentation networks into a classification-like format, enabling the direct use of established attribution methods.
- **Attribution computation:** We adapt and apply attribution methods to produce fine-grained attribution maps from selected layers of the network.

### 3.2. Post-Processing

Visualizing the attribution maps can be a challenging task for several reasons, primarily:

- **Noise and artifacts:** Attribution maps may include noise or irrelevant artifacts inherent from the dataset [49] that can dominate the visualization if not addressed. Therefore, applying normalization and post-processing steps (e.g., smoothing, denoising, or thresholding) helps to highlight the most relevant regions and suppress irrelevant details.
- **Dynamic range and scaling:** Attribution maps often have values in a wide or inconsistent range or very small floating-point values depending on the method and the software implementation used. Ignoring normalization or scaling can obscure patterns, as raw values may not map well to a perceivable range of colors or intensities.

To enhance the comprehension of the attribution maps generated from the first step of our workflow and extract relevant information encompassed inside them, two post-processing steps are introduced: (1) a Clipping step (Section 3.2.1) used to remove outliers, and (2) a Normalization step to distinguish positive and negative attribution and improve their visualization (Section 3.2.2).

#### 3.2.1. Clipping Step

**Figure A1:**
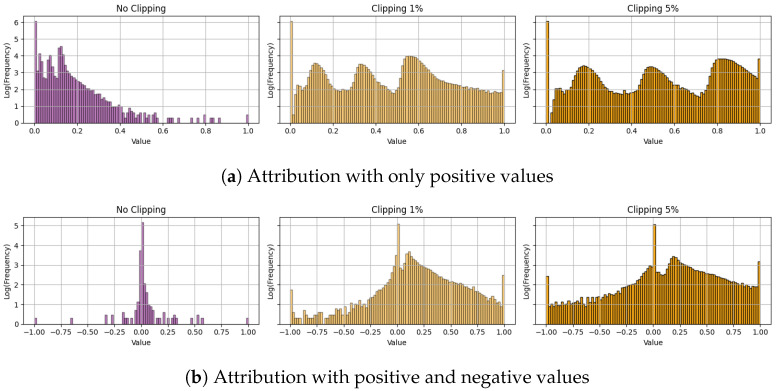
Histogram analysis showcasing the distribution of the attribution values before and after applying the clipping algorithm. The histogram indicates how clipping extreme values with proportions (1% and 5%) reduces the bias caused by outliers while preserving the overall distribution.

**Figure A2:**
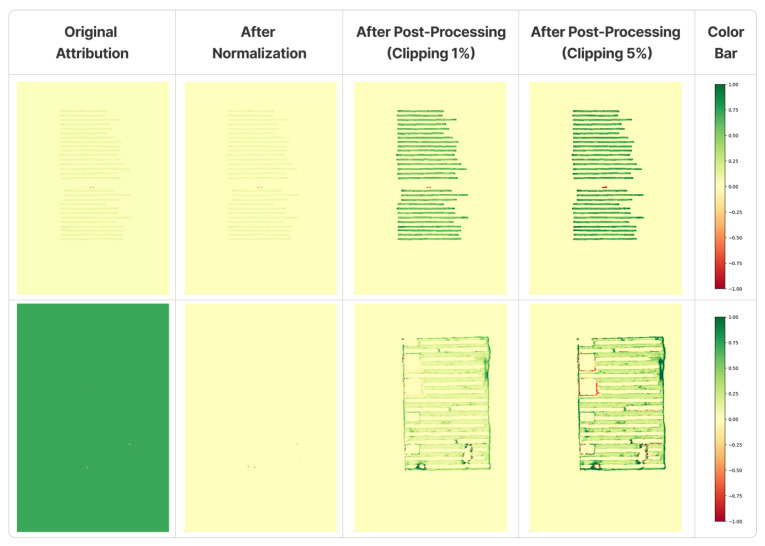
Impact of the refinement step on an attribution map obtained by the LRP method. On each row: from left to right, original attribution without normalization, after applying bipolar normalization, then with introducing the clipping algorithm by setting a 1% and 5% reduction of attributions.

Some generated attribution maps contain exceptional values, leading to imbalances in visualization. Histogram analysis of the attribution values, as shown in Figure A1, indicates a bias toward both higher and lower values, reducing the significance of intermediate values. To tackle this issue, a refinement step is introduced to either substitute or truncate outlier data (see Figure A2).

The clipping algorithm, detailed in Algorithm A2, is designed to enhance the visualization of generated attribution maps by truncating extreme values while preserving the range of intermediate values. It starts first by sorting the input vector of attribution values *V* and identifies thresholds for high and low “outliers” based on a user-defined percentile parameter *p*. Values exceeding these thresholds are replaced with the closest permissible values below or above the thresholds. This step reduces the visual imbalances in the normalized attribution maps, as demonstrated in Figure A2. We observe in Figure A2 hidden attributions when using the clipping algorithm, as it eliminates the large gap in the distribution of values within the normalized attribution maps. The optimal value of the parameter *p* was determined empirically. In what follows, *p* was set to 5% when generating attribution maps.

#### 3.2.2. Normalization Step

Conventional normalization techniques have the potential to alter neutral (zero) values, which can impact the comprehensibility of the data. In this work, we consider three different normalization techniques (symmetric, max-abs, and bipolar range) and analyze their effects through heatmap visualizations and kernel density estimation (KDE) plots.

The symmetric normalization has a major drawback, as any attribution with zero values (neutral values) will shift in position if [a,b] is not symmetrical.

**Figure A3:**
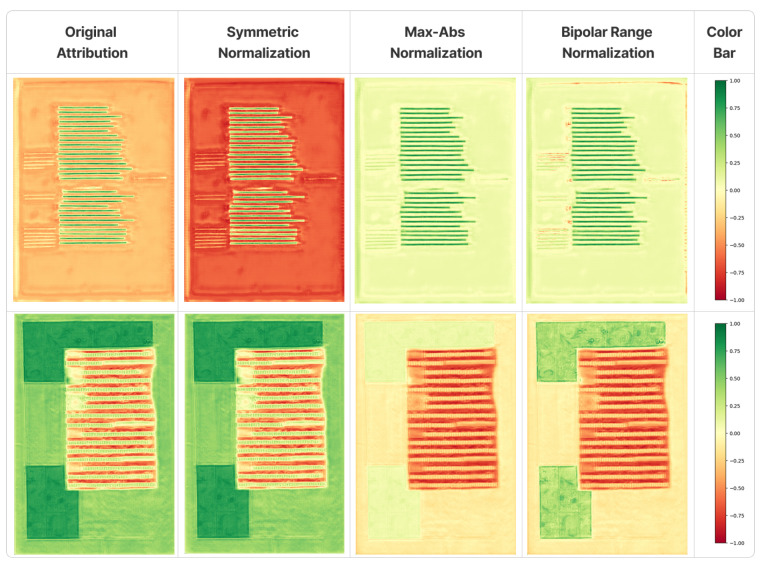
A visual comparison of three normalization techniques applied to two different attribution maps (rows). Each column corresponds to the resulting heatmap before applying any normalization (first column) and after applying a specific normalization method: symmetric (see Equation (A2)), max-abs (see Equation (A3)), and bipolar range (see Equation (A4)). The fifth column shows the heatmap color bar: green for positive attributions, red for negative attributions, and yellow for neutral values.

Figure A3 illustrates the results of normalizing two different attribution maps by means of the three aforementioned techniques (symmetric, max-abs, and bipolar range).

**Figure A4:**
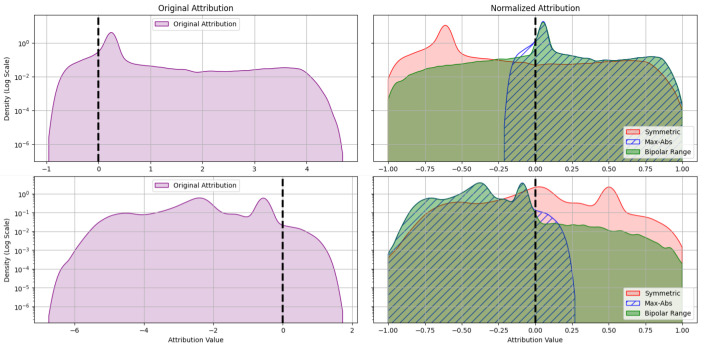
Log-scaled KDE plots of attribution values for two different attributions, displayed row-wise. The first row corresponds to the first attribution, and the second row corresponds to the second attribution. The left column shows the KDE of the original attribution maps before normalization, while the right column compares KDEs of attribution values across three different normalization techniques: symmetric, max-abs, and bipolar range.

A comparison of the first two columns in Figure A3 reveals that the symmetric normalization (first column) differs noticeably from the max-abs normalization (second column). The symmetric normalization fails to maintain neutral attributions, resulting in misleading heatmaps. Using the symmetric normalization, we observe that in the first attribution map (first row), neutral values (yellow) are incorrectly assigned as negative (red), while in the second attribution map (second row), neutral values are misrepresented as positive (green). On the other side, using the max-abs normalization, we note that the neutral values are preserved; however, a visual gap between the positive and negative values appears if the distance between *a* and *b* is relatively large. The KDE plots illustrated in Figure A4 confirm that the density around zero is preserved more accurately with the max-abs normalization compared to the symmetric one.

A comparison of the second and third columns in Figure A3 reveals that, for the first attribution map (first row), negative values (red) are present but less apparent between the green lines in the center when using the bipolar range normalization method (third column). Similarly, for the second attribution map (second row), some positive values (green) are visible only in the third column. The KDE plots in Figure A4 confirm this observation, as the bipolar range normalization method enhances the density of extreme values while retaining neutral attributions.

Based on these observations, bipolar range normalization is the most suitable for our work, as it offers the following advantages:

1. Clear distinction between positive and negative values;
2. Representation of the relationship between target and non-target classes attribution pixels to compare with the ground truth;
3. Improved visuals for qualitative inspection.

### 3.3. Quantitative Evaluation

In the literature, various metrics have been used to evaluate the performance of DNNs dedicated to the layout analysis task. Quantitative evaluation is crucial for objectively assessing the accuracy and limitations of these networks. To measure their performance, we calculated the accuracy and intersection over union metrics. The higher the values of the computed performance evaluation metrics, the better the results.

In addition to visually evaluating the attribution maps, their quality can be objectively measured using dedicated evaluation metrics.

Although XAI-based metrics have been widely explored in the literature, their application to semantic segmentation tasks is still relatively uncommon. In our work, we focus on quantitatively assessing the performance of XAI-based methods across different classes defined in the ground truth for semantic segmentation-based applications. To ensure a fair and constructive comparison between the four XAI methods evaluated in this work, four state-of-the-art evaluation metrics are computed.

### 3.4. Qualitative Evalutation

After quantitatively assessing model performance, we perform a deeper analysis of interpretability by examining attribution maps generated through the SegClarity workflow. The purpose of this evaluation is to measure how effectively input relevance explains the model predictions.

**Figure 4:**
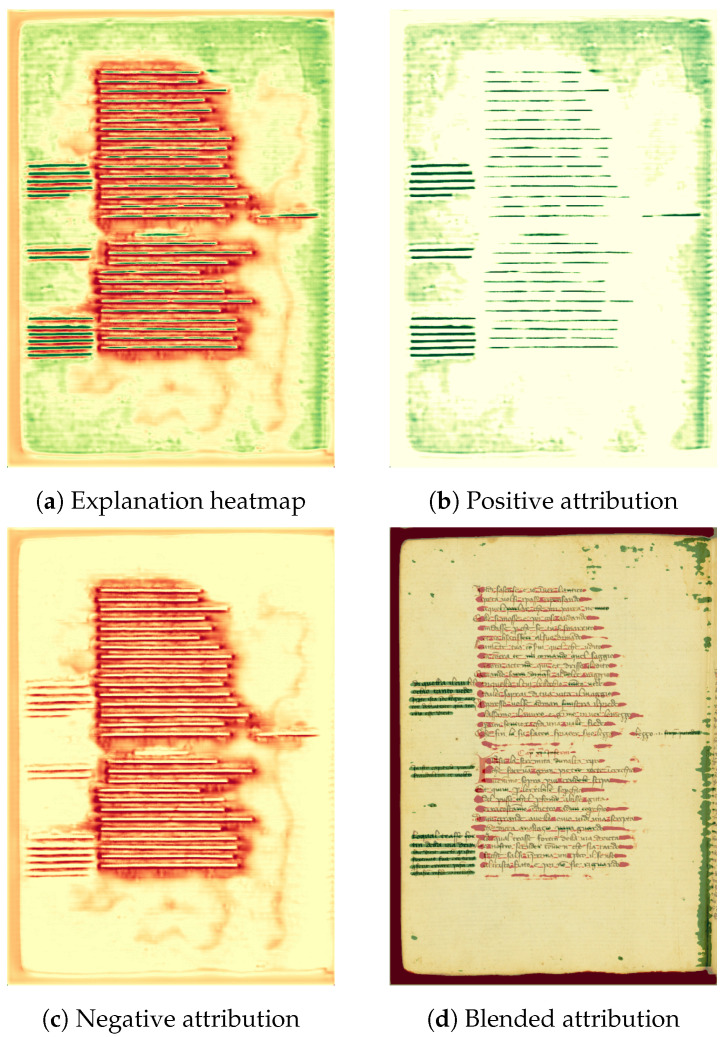
Generated explanations for the *GL* class using Grad-CAM on *Dec2* of the S-U-Net model. (**a**): Complete explanation heatmap; (**b**): Positive attribution heatmap; (**c**): Negative attribution heatmap; (**d**): Blended attribution heatmap superimposed on the input image.

A visual example is illustrated in Figure 4, where heatmaps are used to highlight the contribution of different pixel groups to the final prediction. An association between the input regions and the model prediction is established, which will be further detailed in Section 4.5.3.

### 3.5. Hybrid Evaluation

In this section, we propose a hybrid approach to attribution evaluation that combines qualitative and quantitative methods. It starts by calculating objective scores from evaluation metrics and then performing human-level assessments of generated heatmaps to evaluate how well each metric aligns with human perception. To this purpose, we examine the CH metric by highlighting its limitations, and introduce our novel metric, called the Attribution Concordance Score (ACS). ACS is specifically designed to enhance the robustness of attribution-based explanation assessments. ACS shares the properties of CH and PG, while being tailored to the specifics of our evaluation setting.

#### 3.5.1. Infidelity

Infidelity (Infid) measures the quality of an explanation by quantifying how well it aligns with the predictor response to significant input perturbations. Infid is designed to evaluate the quality of an explanation ϕ(x) for a model *f* at a given input *x*. It specifically quantifies the expected discrepancy between the dot product of the input perturbation and the explanation, and the resulting change in the model output, thereby capturing variations in the *f* values using this formula [22].

(8) $$Infid\left(\phi ,x\right)=\underset{\delta ∼D}{E}\left[{\left(\delta^{⊤}\phi \left(x\right)-\left(f(x)-f(x-\delta )\right)\right)}^{2}\right]$$

where

- ϕ(x) is the explanation map for input *x*;
- δ denotes a perturbation on the input *x* sampled from a distribution *D*;
- f(x) represents the model output on *x*;
- f(x−δ) represents the model output on the perturbed image);
- $\underset{\delta ∼D}{E}$ denotes the expected value over δ.

The concept of fidelity and faithfulness has been recently applied to segmentation tasks [50]. In the context of image modality, we distinguish the target class to interpret cl and the perturbation technique. Infid is adapted to semantic segmentation on a class cl according to the following equation:

(9) $$Infid_{cl}\left(\phi ,x\right)=\underset{\delta ∼D}{E}\left[{\left(\phi_{cl}\left(x\right)⊙\delta_{cl}-\left(f_{cl}\left(x\right)-f_{cl}\left(x-\delta_{cl}\right)\right)\right)}^{2}\right]$$

where

- $\phi_{cl}\left(x\right)$ is the explanation map specific to class label cl for input image *x*;
- $\delta_{cl}$ denotes a perturbation that depends on the input *x*, the label cl, sampled from a distribution *D*;
- $f_{cl}\left(x\right)$ represents the model output for label cl at input *x*, producing a per-pixel confidence score or probability map (i.e., selecting probability map only from target cl);
- $\phi_{cl}\left(x\right)⊙\delta_{cl}$ denotes the element-wise dot product, reflecting the predicted change in the model output according to the explanation $\phi_{cl}\left(x\right)$;
- $\underset{\delta ∼D}{E}$ denotes the expected value over δ.

In our implementation, the perturbation distribution *D* corresponds to a uniform random sampling of square-like localized masks applied over the target class regions (more details in Figure 6). Each perturbation $\delta_{cl}∼D$ is generated by randomly selecting spatial positions and mask sizes within a predefined range, ensuring that every sampled mask occludes a portion of the class-relevant area while preserving the overall image structure.

The Infid values for each individual target class are then aggregated to compute the global Infid score. The selected perturbations $\delta_{cl}$ are applied to the input image *x* through localized noise or occlusions, to more accurately capture the structured nature of segmentation tasks.

The closer the Infid value is to zero, the more accurately the explanation ϕ(x) aligns with the true behavior of the model under perturbations. A lower Infid value indicates that the explanation ϕ(x) reliably captures the model behavior.

**Figure 5:**
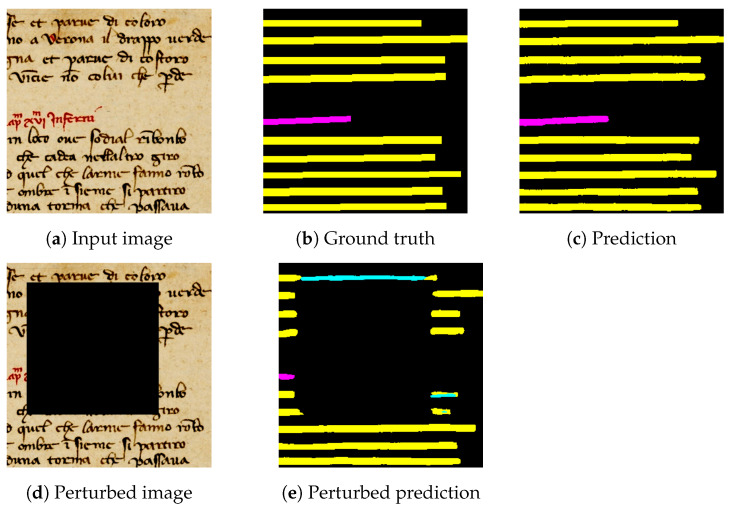
Example of a random square mask perturbation and its impact on segmentation performance. (**a**): Zoomed-in region of the input image; (**b**): Ground truth segmentation mask; (**c**): Model prediction on the original image; (**d**): Perturbed image with a randomly placed square occlusion; (**e**): Prediction on the perturbed image.

Measuring the faithfulness of XAI methods quantifies how accurately an explanation reflects the decision-making process of a DNN and the contribution of input features. Metrics based on ablation are particularly well suited for this purpose, as they systematically remove parts of the input using the perturbation methods and evaluate the resulting impact on the overall interpretation. Common perturbation methods include Gaussian noise, structured noise, or targeted occlusions, chosen based on the specific interpretability task and the characteristics of the input [22]. However, perturbations involving random pixel subsets in high-dimensional images often fail to meaningfully affect model predictions, as minor and unstructured pixel loss is typically compensated for by the surrounding context as shown in Figure 5. This issue becomes particularly critical in tasks involving structured image, where spatial relationships are crucial, such as in document layout analysis.

Figure 5 illustrates the effect of applying a random square perturbation on a localized region of HDI image and its impact on the segmentation maps. In Figure 5, a zoomed-in portion of the input image is shown, focusing on *TXT* and *HL* regions. Figure 5 displays the model prediction on the original image, which correctly segments the main text lines and highlights compared to Figure 5, corresponding to the ground truth. In Figure 5, a square perturbation is applied, occluding a central portion of the input. The prediction on this perturbed image, shown in Figure 5, reveals a significant degradation in segmentation performance. Notably, the occlusion introduces segmentation errors, including the appearance of new, unintended classes, such as *GL*, within the main text area.

In this work, we propose our perturbation method based on semantic annotations, which consists of a square removal technique for generating perturbations, specifically suited to the task of HDI segmentation. We evaluate our method using the Infid metric, which we specifically selected due to its design to work under large perturbations area, as opposed to sensitivity-based metrics that focus on minor input variations.

**Figure 6:**
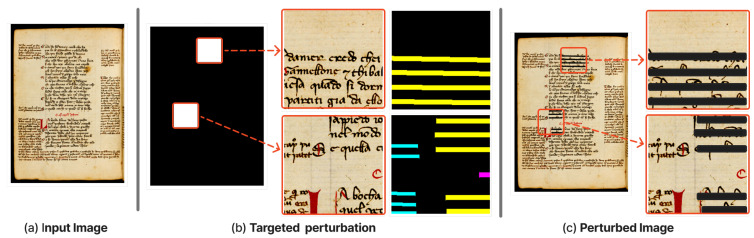
Illustration of the targeted perturbation method applied on HDIs for main text area. (**a**): Original input image; (**b**): Randomly selected square regions for applying targeted perturbations; (**c**): Resulting perturbed image obtained by assigning a black RGB value to the *TXT* pixels defined in the randomly selected square regions.

Our targeted perturbation method is based on constraining perturbations to only pixels that belong to the selected target label, as illustrated in Figure 6. This targeted perturbation method ensures that only the regions relevant to the label of interest are masked, thereby enhancing both the interpretability and relevance of the perturbation on the selected target. By selecting square regions from HDIs, where only the target pixels are considered active, we effectively evaluate the impact on model predictions while preserving the surrounding context. Our square removal technique is a suitable perturbation method for HDI segmentation, particularly in scenarios where regions have semantic significance.

Using the targeted perturbation method, we iteratively generate perturbation masks (i.e., a perturbation mask contains randomly placed square-like patches) until all target classes are covered for all the images of a dataset (see Algorithm 1). The square-like perturbation patches are randomly generated with the condition that each target class present in the input (i.e., where pixels are non-zero) is properly represented in the perturbation mask. Our method ensures that the perturbed regions contain at least a group of pixel from each class in the set of target classes *T*.
**Algorithm 1** Procedure1:**Input:** *X * (input image), *T* (set of target classes), GT (ground truth mask)2:**Output:** *M * (perturbation mask containing multiple square patches and covering all target classes present in *X*)3:**repeat**4:   M←GenerateTargetedPerturbationMask(X)5:   $C\leftarrow \{GT_{i,j}|M_{i,j}=1\}\cap T$6:**until** C=T**return** *M*

#### 3.5.2. Sensitivity

Sensitivity maximum (Sens_Max) measures the robustness of an explanation by evaluating how much it changes in response to small variations in the input. Sens_Max, specifically, focuses on the maximum change in the explanation. It identifies the largest deviation in the explanation, highlighting features or areas most affected by input perturbations [22].

Formally, Sens_Max for an explanation Φ of a model *f* at an input *x* with a neighborhood radius *r* is defined as

(10) $$Sens\_Max\left(\Phi ,f,x,r\right)=\max_{|\epsilon |<r}∥\Phi \left(f,x+\epsilon \right)-\Phi \left(f,x\right)∥$$

where

- ϵ denotes a perturbation vector applied to the input *x*, with |ϵ|<r constraining it to a neighborhood of radius *r* around *x*;
- Φ(f,x) is the explanation map generated by the explanation functional Φ for the model *f* at the input *x*;
- Φ(f,x+ϵ) is the explanation produced for the perturbed input x+ϵ, providing insight into the model sensitivity to local changes;
- ∥·∥ represents a norm (commonly the $L_{2}$-norm), reflecting the magnitude of the difference in the explanation values.

Sens_Max captures the peak response of the explanation to perturbations, indicating how stable or robust the explanation is under local changes in the input.

A low Sens_Max value suggests that the explanation remains relatively consistent even when the input is slightly altered, which is desirable for trustworthy and interpretable models. In contrast, a high Sens_Max value may indicate that the explanation is highly sensitive to small changes, potentially reducing the reliability of the interpretability of the adopted XAI-based method.

#### 3.5.3. Pointing Game

Pointing game (PG) evaluates the precision of attribution maps by checking whether their most salient point falls within the ground truth region of the target class. Unlike overlap-based metrics, PG focuses on the single pixel with the highest attribution score and assesses whether this “pointing” corresponds to a semantically meaningful location.

A higher PG score indicates that the attribution method successfully highlights the most relevant region for the target class.

In our work, we adopt the PG metric following the formulation introduced by Zhang et al. [24]. Originally proposed for weakly supervised object localization, we extend this approach to semantic segmentation, enabling pixel-level evaluation of attribution methods.

$PG_{cl}$ for a target class cl is defined according to the following equation:

(11) $$PG_{cl}=\left\{\begin{array}{cc} 1, & \mathrm{if}\;\arg\max_{i,j}A_{ij}\in \left\{\left(i,j\right):M_{cl_{ij}}=1\right\},\\ 0, & \mathrm{otherwise}, \end{array}\right.$$

where

- $PG_{cl}$ represents the PG score for class cl;
- $A_{ij}$ is the attribution value at pixel (i,j);
- $M_{cl_{ij}}$ is a binary mask indicating whether pixel (i,j) belongs to class cl.

The overall PG score is defined as the mean over *N* evaluated samples, computed as

(12) $$PG=\frac{1}{N}\sum_{n=1}^{N}PG_{cl}^{\left(n\right)}.$$

#### 3.5.4. Content Heatmap

The content heatmap (CH) measures the overlap between the attribution values and the segmentation mask, enabling a more contextually relevant evaluation of model explanations. It works by measuring the proportion of attribution values that fall within the regions corresponding to the target class, effectively capturing the contextual relevance of the explanation. A higher CH score indicates that the model focuses its attention on semantically meaningful regions when making predictions. In our work, we use the CH metric by leveraging the entire segmentation map, following the approach proposed by Wand et al. [51]. This approach provides a novel perspective, differing from previous applications of CH that were not tailored to segmentation-based applications.

(13) $$CH_{cl}=\frac{\sum_{i,j}A_{ij}\times M_{cl_{ij}}}{\sum_{i,j}M_{cl_{ij}}}$$

where

- $CH_{cl}$ represents the CH value for class cl;
- $A_{ij}$ is the attribution value at pixel (i,j);
- $M_{cl_{ij}}$ is a binary mask indicating whether pixel (i,j) belongs to class cl.

#### 3.5.5. Proposed ACS Metric

CH has been used to evaluate XAI methods by quantifying the proportion of heatmap regions overlapping with the ground truth mask of the target object relative to the entire heatmap (see Section 3.4).

Nonetheless, it suffers from a major limitation: attribution values falling outside the boundaries of the target class are disregarded. To illustrate this shortcoming, we construct a forged attribution map $A_{F}$ (see Equation (16)) from an original attribution map *A* generated using the proposed workflow (see Section 3). In $A_{F}$, the heatmap of the target region (*T*) remains visually unchanged, while the heatmap outside the target region is altered. The forged attribution map is computed as follows:

(14) $$\chi_{T}\left(x\right)=\left\{\begin{array}{cc} 1, & \mathrm{if}\;x=T\\ 0, & \mathrm{otherwise} \end{array}\right.$$

(15) $$M_{T}\left(A\right)={\left(\chi_{T}\left(A\left(i,j\right)\right)\right)}_{i\leq n,\;j\leq m}$$

(16) $$A_{F}=A⊙M_{T}\left(A\right)+N_{\left[0,1\right]}\left(A⊙M_{O\neq T}\left(A\right)\right)\cdot {∥A⊙M_{T}\left(A\right)∥}_{\infty }$$

$M_{T}\left(A\right)$ isolates target pixels *T*, $M_{O\neq T}\left(A\right)$ isolates non-target regions, and $N_{\left[0,1\right]}$ normalizes the irrelevant heatmap outside *T*. The normalization ensures consistent visual comparison after modifying non-target attributions.

**Figure A5:**
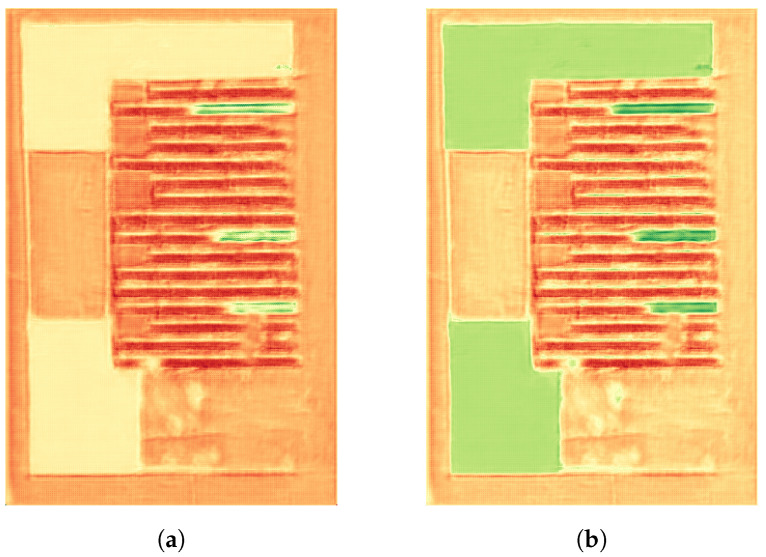
Comparison of the original and forged attribution maps. Despite visual differences, both attribution maps yield identical CH values, highlighting the failure to penalize attributions outside the target class. (**a**) Attribution with GradCAM, CH=0.574|ACS=0.010. (**b**) Forged attribution, CH=0.574|ACS=0.030.

Figure A5 illustrates two different attribution maps, the original attribution map generated by GradCAM (see Figure A5) and the forged attribution map (see Figure A5). Both attribution maps have identical CH values, underscoring the inability of CH to penalize attributions that fall outside the boundaries of the target class. This highlights the need for an optimized evaluation metric that accounts for attribution distributions both within and beyond the target boundaries. To overcome this limitation, we introduce in this work the ACS metric, a metric designed to evaluate attribution maps more comprehensively. An effective attribution map should fulfill several key criteria. In the context of a target class *T*, it is expected that the positive attributions within the attribution map will mostly emphasize pixels that are associated with class *T*, while the negative attributions should primarily emphasize pixels that are not associated with class *T*.

**Figure 7:**
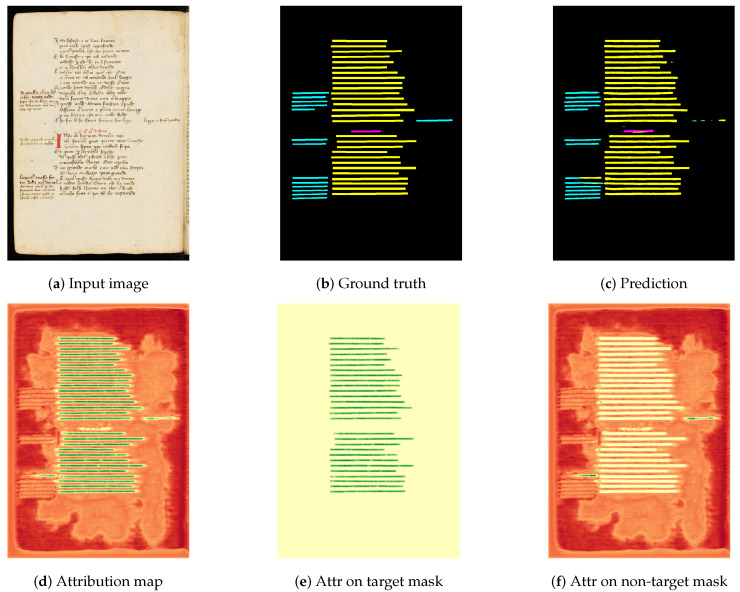
Illustration of the ACS metric computation. It begins with the original input image (**a**), followed by the annotated ground truth (**b**) and the model predictions (**c**). An attribution map is then generated (**d**), which is subsequently overlaid on the target regions (*TXT*) (**e**). Finally, the attribution is disentangled from non-target regions, specifically the *BG*, *GL*, and *HL* classes (**f**).

To implement the ACS metric, as illustrated in Figure 7, we start by extracting the attribution maps and generating a binary mask that represents class *T*. In this mask, pixels that belong to class *T* are assigned a value of 1, while pixels that are not part of *T* are assigned a value of 0. Then, the mask is applied on the attribution maps, pixel-wise multiplication, resulting in a new attribution map referred to the attribution on target class $A_{T}$. This map highlights the attributions that are directly associated to the target class. On the other hand, by the inversion of values in a mask, another attribution map is generated, referred to the attribution on non-target class $A_{\bar{T}}$. This map includes attributions that are not linked to the target class. Our objective is to ensure that positive attributions are primarily represented in $A_{T}$, while negative attributions are more concentrated in $A_{\bar{T}}$. To formalize this analysis, we introduce terminology that distinguishes between true positives and false negatives in $A_{T}$, and false positives and true negatives in $A_{\bar{T}}$. By leveraging this terminology, we compute conventional evaluation metrics, such as precision, recall, accuracy, and F1-score. To enhance the robustness of attribution-based explanation assessments, we propose the ACS metric. ACS can be seen as the F1-score counterpart in our attribution-based XAI workflow.

Given a model *M* and an attribution map *A*, we first decouple *A* into two different components:

1. Attribution on target mask, defined as
  (17) $$A_{T}={\left(\chi_{T}\left(A\left(i,j\right)\right)\right)}_{i\leq n,j\leq m}$$
  where χ is the indicator function defined in Equation (14).
2. Attribution on non-target mask, defined as
  (18) $$A_{\bar{T}}=A-A_{T}$$

Second, we compute the following measures:

$$\begin{array}{cccc} TP & =\sum_{i,j}\max\left(0,A_{T}\left(i,j\right)\right) & & (\mathrm{True}\;\mathrm{Positive});\\ FN & =\sum_{i,j}\min\left(0,A_{T}\left(i,j\right)\right) & & (\mathrm{False}\;\mathrm{Negative});\\ FP & =\sum_{i,j}\max\left(0,A_{\bar{T}}\left(i,j\right)\right) & & (\mathrm{False}\;\mathrm{Positive});\\ TN & =\sum_{i,j}\min\left(0,A_{\bar{T}}\left(i,j\right)\right) & & (\mathrm{True}\;\mathrm{Negative}). \end{array}$$

After computing TP, FN, FP, and TN, we calculate the two following evaluation metrics: precision (P) (see Equation (19)) and recall (R) (see Equation (20)).

(19) $$\mathrm{Precision}=\frac{\mathrm{TP}}{\mathrm{TP}+\mathrm{FP}}$$

(20) $$\mathrm{Recall}=\frac{\mathrm{TP}}{\mathrm{TP}+\mathrm{FN}}$$

Based on the precision and recall metrics, we propose the ACS metric, as defined in Equation (21). ACS corresponds to the harmonic mean of precision and recall. Its formulation is similar to that of the F1-score metric.

(21) $$ACS=2\times \frac{\mathrm{Precision}\times \mathrm{Recall}}{\mathrm{Precision}+\mathrm{Recall}}$$

#### 3.5.6. Adaptive ACS Metric

**Figure A6:**
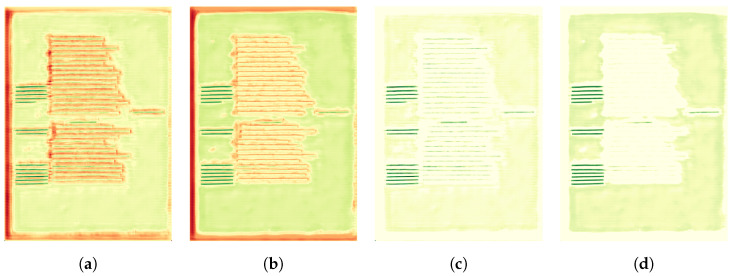
Limitation of the ACS metric illustrated when using the proposed workflow by setting different model layers with GradCAM and *GL* target class. (**a**) GradCAM, *Dec3*, CH=0.644, ACS=0.240. (**b**) GradCAM, *Dec1*, CH=0.689, ACS=0.185. (**c**) GradCAM + ReLU, *Dec3*, CH=0.480, ACS=0.241. (**d**) GradCAM + ReLU, *Dec1*, CH=0.556|ACS=0.185.

ACS is well suited to the context of our work and yields a more accurate evaluation than CH; however, there are cases based on qualitative assessment of some attribution maps that prove a misalignment with the quantitative evaluation, as illustrated in Figure A6. The low ACS score can be explained by the distribution of the positive attributions over non-target classes, which reduces the precision of the ACS metric. In the first example in Figure A6, the positive attributions are concentrated on the target class *GL*, while other lower values positive attributions (in light green) are shown on the background pixels. Each of the four examples illustrates positive attribution of the target class *GL*, though affected to some extent by the background class.

To address this issue, we propose a thresholding step that enhances the saliency of key attribution areas and decreases the impact of noise from less significant attribution pixels. The thresholding step is based on using the Otsu method [52]. The Otsu method is a thresholding technique used widely in image processing to separate the foreground from the background. It works by finding the threshold that minimizes the intra-class variance, or equivalently maximizes the inter-class variance. The Otsu threshold $t^{*}$ corresponds to the value of *t* that maximizes $\sigma_{B}^{2}\left(t\right)$. $\sigma_{B}^{2}\left(t\right)$ is defined according to the following equation:

(22) $$\sigma_{B}^{2}\left(t\right)=\omega_{0}\left(t\right)\omega_{1}\left(t\right){\left[\mu_{0}\left(t\right)-\mu_{1}\left(t\right)\right]}^{2}$$

where

- $\sigma_{B}^{2}\left(t\right)$ is the between-class variance at threshold *t*;
- $\omega_{0}\left(t\right)$ and $\omega_{1}\left(t\right)$ are the probabilities of the two classes separated by the threshold *t*;
- $\mu_{0}\left(t\right)$ and $\mu_{1}\left(t\right)$ are the means of the two classes separated by the threshold *t*.

In our work, the Otsu method is applied to determine optimal thresholds from the attribution maps. These thresholds are subsequently used to normalize the attribution maps, thereby improving the precision of the ACS metric. For this purpose, we adopt the following pipeline:

1. Divide the attribution map into two components: positive attributions (A+) and negative attributions (A−);
2. Perform the Otsu method on A+ to determine the threshold $th_{+}$ and retain values exceeding this threshold;
3. Generate $\tilde{A+}$ by assigning the threshold $th_{+}$ to the values in A+ that exceed $th_{+}$;
4. Perform the Otsu method on A− to determine the threshold $th_{-}$ and retain values less than this threshold;
5. Generate $\tilde{A-}$ by assigning the threshold $th_{-}$ to the values in A− that exceed $th_{-}$;
6. Merge the refined attribution maps $\tilde{A+}$ and $\tilde{A-}$ to obtain the refined attribution map $\tilde{A}$.

**Figure 8:**
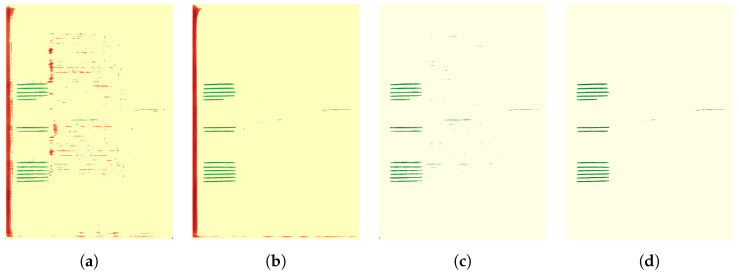
Comparative visualizations of XAI methods after applying the thresholding method on attribution maps. (**a**) GradCAM, *Dec3*, CH=0.430, ACS=0.914. (**b**) GradCAM, *Dec1*, CH=0.503, ACS=0.969. (**c**) GradCAM + ReLU, *Dec3*, CH=0.430, ACS=0.914. (**d**) GradCAM + ReLU, *Dec1*, CH=0.503, ACS=0.969.

Figure 8 illustrates the effect of applying the thresholding method to the attribution maps and its impact on the ACS metric. We observe that the quality and interpretability of the visual outputs are enhanced, and the computed ACS metric aligns well with the visual results.

#### 3.5.7. Human Assessment

Our main objective is not only to evaluate the effectiveness of an XAI-based method, but also to determine the level of comprehension of the model (i.e., plausibility). This corresponds to measuring the model’s ability to capture understanding and its alignment with human expectations or domain knowledge. The evaluation phase was divided into two parts. For the historical document datasets, the assessment was conducted by domain experts specializing in document analysis—primarily researchers and Ph.D. holders in information extraction from visually rich documents, historical document image analysis, and text-line segmentation of ancient manuscripts, including members of the https://iapr.org/ (access on 8 November 2025). For the Cityscapes dataset, the evaluation was carried out by Ph.D. candidates and engineering students with expertise in image processing, particularly in aerial imagery object detection and hand gesture recognition. To ensure objectivity and eliminate potential bias, none of the authors participated in the evaluation process. Hence, we introduce an evaluation protocol for human assessment of heatmaps, assigning scores according to the specific scenario:

- **High score (✓✓):** In cases where the heatmap assigns positive attribution to the pixels that represent the class of interest;
- **Medium score (✓):** In cases where the heatmap does not assign positive attribution to all pixels of the class of interest or it assigns positive attribution to the pixels of the relevant class and only one additional class;
- **Low score (×):** In cases where the heatmap does not assign positive attribution to the pixels that represent the target class.

**Figure 9:**
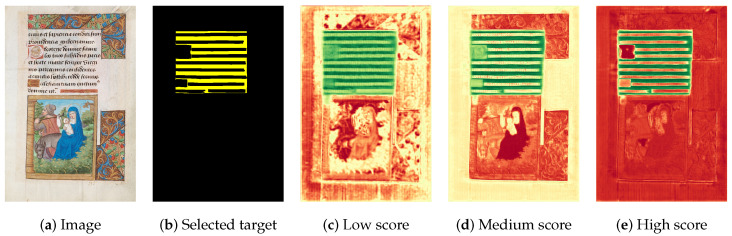
Illustration of the human-based scoring system. (**a**): input image from UTP-110; (**b**): selected target class (BD) in yellow; (**c**): low score with dispersed attributions; (**d**): medium score with attributions on BD and partly on BInit; (**e**): high score with attributions concentrated on BD.

Figure 9 illustrates the three human-based scoring scenarios. From an input image in the UTP-110 dataset (see Figure A10), we selected the target class body (BD), highlighted in yellow. In the first case (see Figure 9), the positive attributions in green are dispersed across multiple classes, resulting in a low score. In the second case (see Figure 9), most positive attributions concentrate on the BD class, although another class (BInit) also shows a noticeable response, which corresponds to a medium score. Finally, in the third case (see Figure 9), the positive attributions are strongly concentrated on the BD class, while the other classes remain neutral or negative, indicating a high score.

Among the five computed metrics, three are particularly aligned with human assessments: CH, ACS, and PG, as they rely solely on heatmap analysis. In contrast, Infid and Sens_Max depend on perturbations. Further experiments and analyses of these metrics are presented in Section 4.6.

## 4. Experiments and Results

To showcase the applicability of the proposed workflow and the different contributions, we conduct a set of thorough experiments on two types of datasets: real and artificial, which we describe in detail in the following sections.

### 4.1. Experimental Corpora

**Figure 10:**
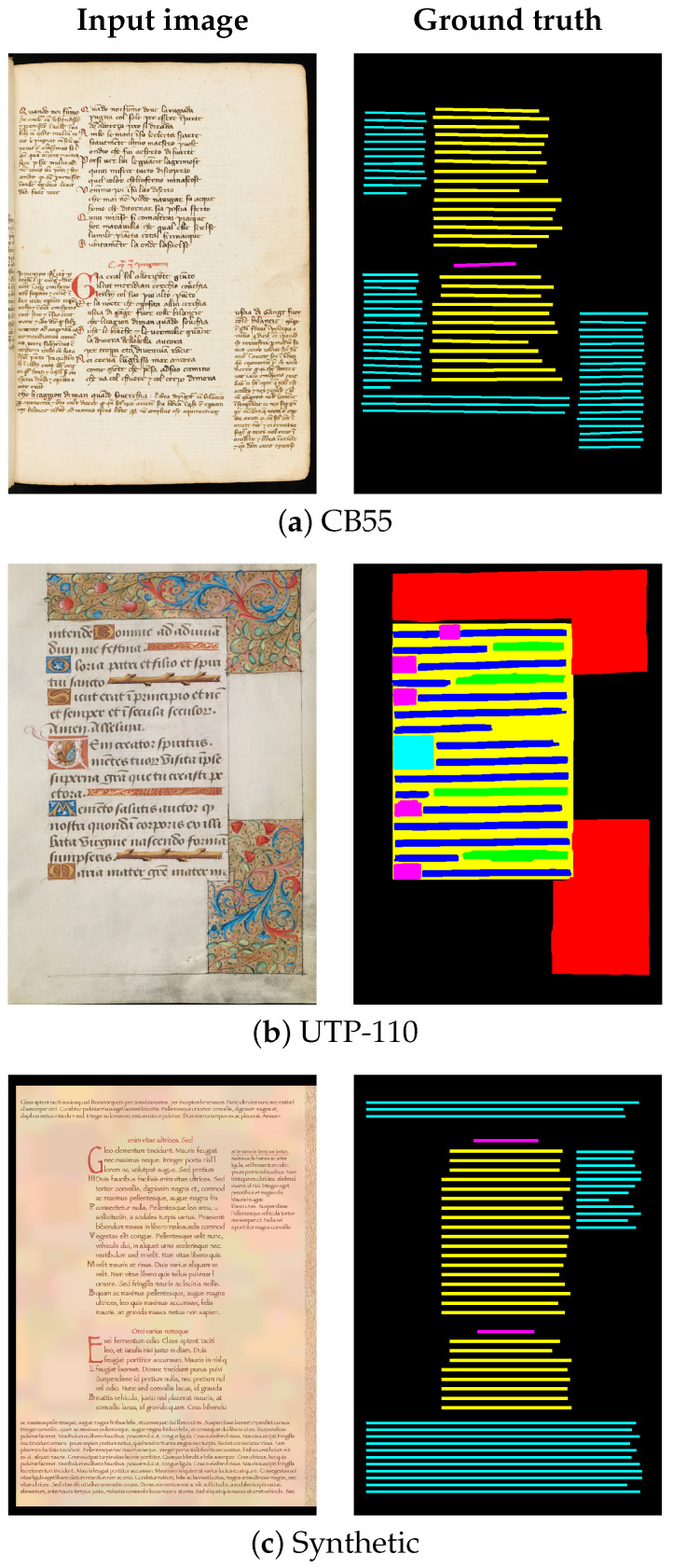
Examples of historical document images and their corresponding ground truth masks used in our experiments. (**a**) CB55 dataset, (**b**) UTP-110 dataset, and (**c**) Synthetic dataset. In the CB55 and Synthetic ground truth masks, black, yellow, cyan, and magenta represent background (*BG*), main text (*TXT*), glosses (*GL*), and highlight (*HL*), respectively. In the UTP-110 ground truth, black, red, yellow, blue, cyan, magenta, and green represent background (*BG*), decoration (*DEC*), body (*BD*), text line (*TXTL*), big initial (*BInit*), small initial (*SInit*), and filler (*FIL*).

To analyze the performance of the four evaluated XAI-based methods on the two considered models, we conducted experiments based on both qualitative and quantitative observations derived from HDI collected from various benchmark datasets dedicated to document layout analysis. These datasets were provided in the context of recent open competitions at the ICDAR and ICFHR conferences. Consequently, our experimental corpus consists of the following three datasets:

1. **CB55 dataset** (https://diuf.unifr.ch/main/hisdoc/diva-hisdb.html) (accessed on 8 November 2025): This is a freely available subset of the DIVA-HisDB dataset containing Medieval manuscripts. It is composed of 70 Latin handwritten document images digitized at 600 dpi (see Figure 10a). Four classes are defined in the ground truth column: *TXT* (i.e., central text), *HL* (i.e., a special line separating paragraphs in main text), *GL* (i.e., text on the page sides), and *BG*. The CB55 dataset presents various particularities (e.g., decorations and comments written in different calligraphy styles) [19].
2. **UTP-110 dataset:** This is a subset of Medieval manuscripts (collection Utopia, armarium codicum bibliophilorum, Cod. 110) [20]. It contains 300 images, primarily in Latin with some sections in French (see Figure 10b). The UTP-110 images were resized to 640×960 pixels while preserving the original aspect ratio. Seven classes are defined in the ground truth, as shown in Figure 10: background (*BG*), decoration (*DEC*), body (*BD*), text line (*TXTL*), big initial (*BInit*), small initial (*SInit*), and filler (*FIL*). The UTP-110 dataset presents complex challenges for layout analysis, including various types of ornaments, decorative text, faded writing, and ink bleed-through [20].
3. **Synthetic dataset:** This is a collection of 150 synthetic HDIs designed with fonts and layouts that capture the key characteristics of the CB55 dataset (see Figure 10c). To ensure adequate variability, three different fonts with distinct sizes are used [12].

The two real datasets, CB55 and UTP-110, provide complementary data sources to evaluate and enhance the performance of DNNs across multiple classes and resolutions.

### 4.2. Experimental Protocol

In our experiments, we used the ground truth defined at pixel level to assess the performance of two deep architectures dedicated to semantic segmentation. Our experiments focus on two U-NET variants:

- **S-U-NET:** This is the standard U-NET version featuring a high number of channels in each decoder block (512, 256, 128, and 64, from the bottleneck to the segmentation head), comprising over 31 million parameters [25].
- **L-U-NET:** This is a lightweight version of the standard U-NET introduced by Rahal et al. [12], which has only 16 channels in each decoder block and fewer than 17,000 parameters.

L-U-NET and S-U-NET architectures follow the same structural design, differing only in the depth and number of channels. For instance, L-U-NET has a single convolution layer in each block, whereas S-U-NET includes two convolution layers per block.

The training process for both two U-NET variants was carried out in two stages on the CB55:

1. **Pre-training** was performed using the synthetic dataset, during which the model with the lowest validation loss was selected;
2. **Fine-tuning** was performed using the best performing model on the real datasets of CB55.

For the UTP-110 dataset, both models were trained directly on the real dataset without a pre-training phase.

In our experiments, we initially pre-trained the L-U-NET and S-U-NET models using a synthetic dataset to address the challenge posed by the limited number of training pixels in the CB55 dataset, particularly for the *HL* class. The synthetic dataset consists of 150 images, divided into 120, 20, and 10 for training, testing, and validation, respectively.

After pre-training, we fine-tuned the two models (L-U-NET and S-U-NET) on the CB55 dataset, which contains 70 images divided into 40, 20, and 10 for training, testing, and validation, respectively. We also fine-tuned the two deep models on the UTP-110 dataset, which comprises 100 images, divided into 60, 20, and 20 for training, testing, and validation, respectively. The two U-NET architectures were trained with a batch size of 5, using the ADAM optimizer that was configured with epsilon and learning rate values of $10^{-5}$ and $10^{-3}$, respectively. Cross-entropy was adopted as the loss function for training both U-NET architectures. All the experiments were performed on a Linux server equipped with 8 Tesla V100-SXM2 GPUs (32 GB memory each), 64 cores, and 755 GB RAM. Our training strategies were implemented using the *PyTorch (version 2.0.1)* and *Captum frameworks (version 0.7.0)* and executed on an *Nvidia RTX 3060 GPU* with 16 cores and 16 GB RAM.

### 4.3. Explanation Parameters

**Figure A7:**
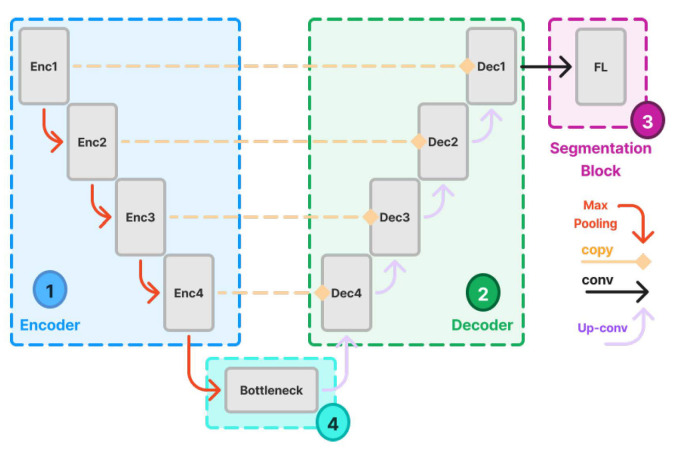
Overview of the U-NET architecture, highlighting the encoder, bottleneck, and decoder blocks, along with the final layer (FL).

To generate attribution maps, we focus on five stages of the U-NET architecture. Specifically, we analyze the decoder part, as depicted in Figure A7. The decoder blocks (*Dec4* to *Dec1*) are paired with corresponding encoder blocks via skip connections, while the bottleneck serves as the transition between the encoder and decoder. The segmentation head at the end of the architecture which corresponds to the final layer (*FL*), generates the final output.

We chose the final layer of each decoder block as it provides the most semantically informative feature representations. Earlier decoder layers often capture intermediate, task-specific transformations that are difficult to interpret independently. By combining upsampled features with skip-connected encoder outputs, the final decoder layer achieves a balance between semantic context and spatial detail. Accordingly, we focus on the final layer of each decoder block and the ultimate segmentation head.

To ensure a fair comparison of the explainability measures between the two U-NET variants, we limited our analysis to the first convolutional layer in each block.

All the attribution-based XAI methods are capable of generating both positive and negative attribution maps. Hence, in our work, we used a version of Grad-CAM without the ReLU layer, allowing negative attributions to be visualized.

Additionally, we note that each layer generates attribution maps with different spatial dimensions, as each attribution is tied to the parameters of its corresponding layer. As depicted in Table 2, lower layers have smaller dimensions compared to higher layers. For instance, *Dec1* and *FL* spatial dimensions are 16 larger than *Dec3* and 64 larger than *Dec4*. For visual comparison, we applied image interpolation to the resulting heatmaps to ensure that they align with the same scale.

For attribution-based XAI evaluation, the Infid and Sens_Max metrics were computed using 10 random perturbations per image. All attribution-based methods were evaluated with batches of 3 images, except for S-U-NET with LRP, which was restricted to a batch size of 1 due to memory constraints. The input neighborhood radius *r* was set to 0.2, following the approach of Yeh et al. [22]. Although Yeh et al. [22] noted that varying *r* can affect Sens_Max outcomes, exploring this parameter within the segmentation context is computationally prohibitive, as shown in Table 7.

**Figure 11:**
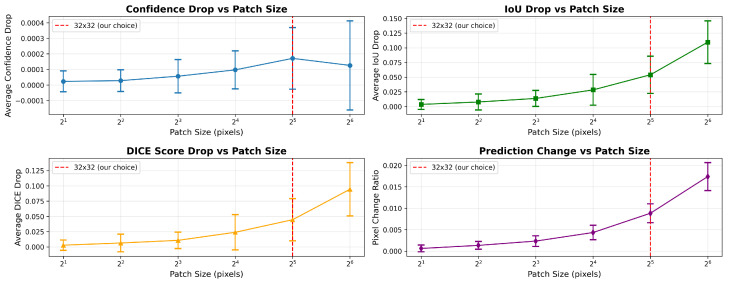
Impact of perturbation mask size on infidelity sensitivity. Six patch sizes (2 × 2 to 64 × 64) were tested across 50 random patches and 100 iterations.

For the perturbation mask generation, we performed a mask-size sensitivity analysis to chose the optimal mask size for the experiment. This experiment validates the choice of 32 × 32 patch size for the infidelity metric by systematically testing different mask sizes (2×2,4×4,8×8,16×16,32×32,64×64) and measuring their effect on model performance using model Confidence Drop, IoU Drop, DICE Drop, and Pixel Change Ratio. We fixed a L-UNET model on CB55 and averaged over 100 iterations and 50 random patches. The results are shown in Table 3 and Figure 11. Larger masks (>64×64) produce excessive degradation due to over-occlusion, whereas smaller ones (<16×16) yield minimal response variation. The 32×32 mask provides an optimal trade-off between perturbation strength and attribution precision.

### 4.4. Generalization Setup

To evaluate the domain-specific behavior of explainable segmentation methods, we conducted a comparative experiment using six XAI techniques: four gradient- and decomposition-based methods (*Grad-CAM*, *Gradient × Input*, *LRP*, and *DeepLIFT*) and two perturbation-based methods (*RISE* and *MiSuRe*). The experiment was first performed on two historical document datasets, CB55 and UTP-110, focusing on the main text class to analyze how each method highlights semantically relevant regions. The main drawback of the perturbation method is the inability to use the intermediate layer; therefore, for this experiment, we only consider explaining the input layer of the network. The results show that perturbation-based methods, particularly RISE, failed to produce meaningful attributions in document images due to the complex background textures and sparse textual structures, while MiSuRe yielded overly generalized activations. In contrast, the other attribution-based methods generated precise and semantically coherent explanations aligned with textual regions. To further validate the robustness and generalization of the framework, we repeated the same analysis on the Cityscapes dataset, which represents a fundamentally different visual domain. In this context, both perturbation- and attribution-based methods produced consistent and interpretable heatmaps. Table 4 summarizes the experimental configurations used for the perturbation-based methods, with parameters empirically tuned to balance mask resolution, sparsity, and computational stability across datasets.

### 4.5. Results

**Figure 12:**
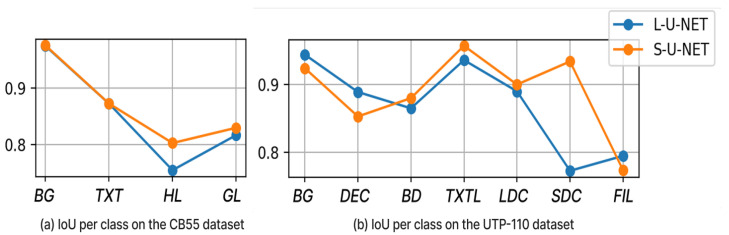
Comparison of performance using the IoU metric between the L-U-NET and S-U-NET models on the CB55 and UTP-110 datasets for each individual target class.

**Figure A8:**
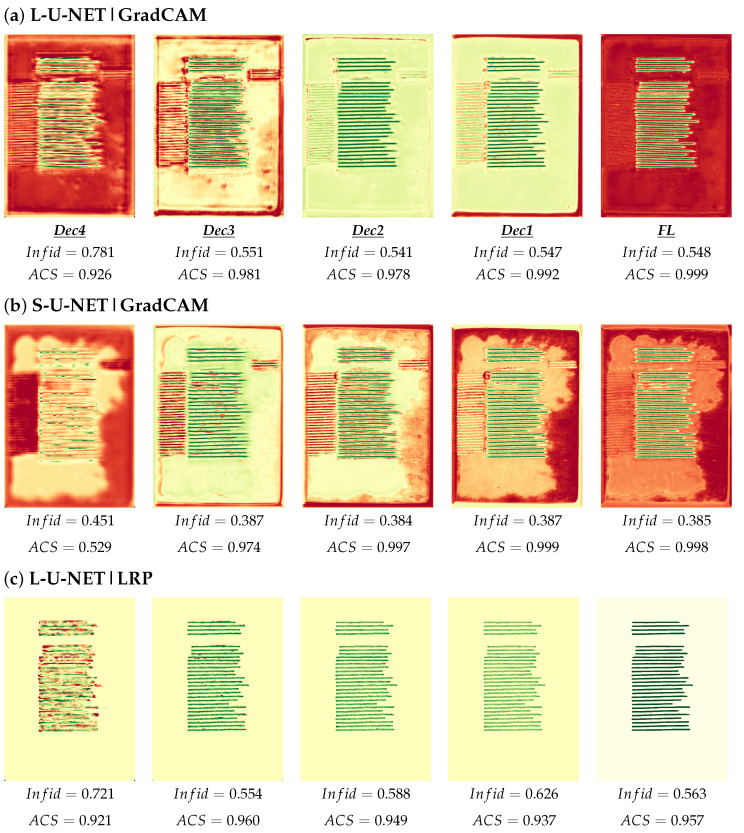

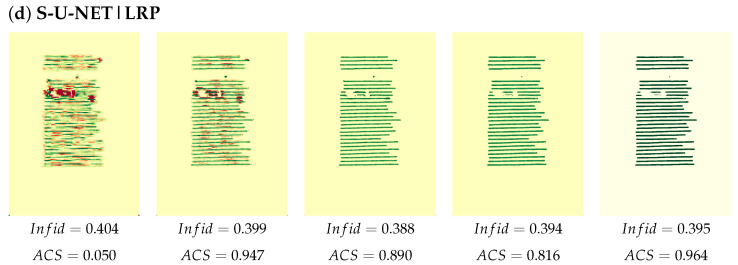
Attribution maps for decoder layers (*Dec4* to *FL*) in L-U-NET and S-U-NET, evaluated on the CB55 dataset targeting main text pixels (*TXT*). Rows (**a**,**b**) show GradCAM; rows (**c**,**d**) show LRP. Infid and ACS are displayed below each panel to quantify attribution quality across layers and methods.

**Figure A9:**
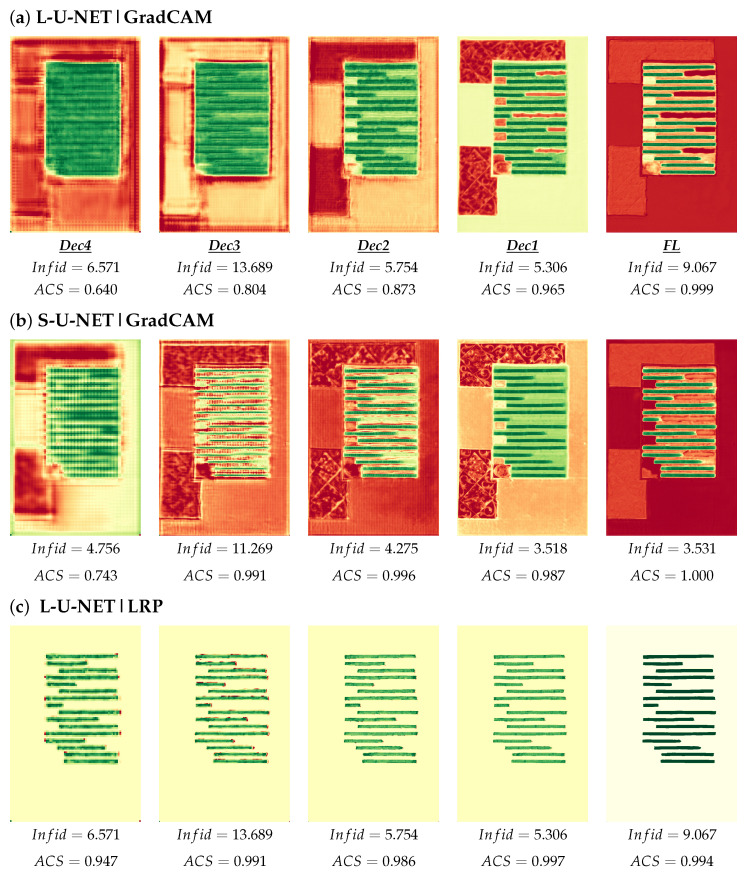

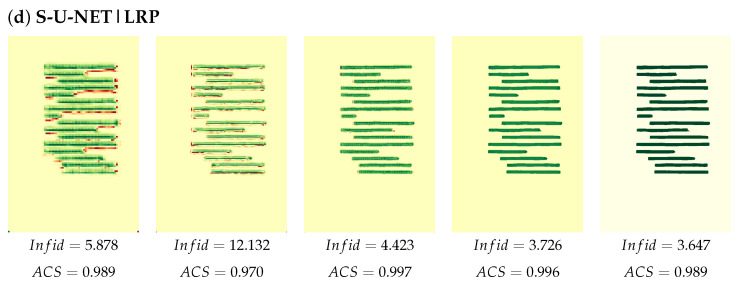
Attribution maps for decoder layers (*Dec4* to *FL*) in L-U-NET and S-U-NET, evaluated on the UTP-110 dataset targeting text line pixels (*TXTL*). Rows (**a**,**b**) use GradCAM; rows (**c**,**d**) use LRP. Infid and ACS are shown below each panel.

This subsection reports both predictive performance and explainability outcomes across datasets and layers. We first benchmark the two U-NET variants (L-U-NET and S-U-NET) on the CB55 (four classes) and UTP-110 (seven classes) datasets using standard segmentation metrics, including accuracy, precision, recall, F1-score, and intersection over union (IoU), which are summarized in Table 5 and detailed per class in Figure 12. We then evaluate the quality of the generated attribution maps across five layers (*Dec4* → *Dec1* and *FL*) using four different XAI metrics, including infidelity (Infid), sensitivity (Sens_Max), content heatmap (CH), and the proposed Attribution Concordance Score (ACS)—aggregated as overall averages (*A*) and foreground-only averages (fA) in Table A1 and Table A2. Finally, we qualitatively analyze layer-wise attribution patterns with Grad-CAM and LRP on representative samples (see Figure A8 and Figure A9), relating visual trends to the quantitative scores.

#### 4.5.1. Model Performance

The quantitative evaluation was conducted separately on the CB55 and UTP-110 datasets, which contain 4 and 7 target classes, respectively, across 5 layers (*Dec4* to *Dec1* and *FL*), as described in Section 4.3.

As shown in Table 5, S-U-NET outperforms L-U-NET in both IoU and F1-score across the CB55 and UTP-110 datasets, while L-U-NET maintains a relatively comparable performance despite having fewer parameters.

Figure 12 illustrates the comparison of performance using the IoU metric between the L-U-NET and S-U-NET models on the CB55 and UTP-110 datasets for each individual target class. For the CB55 dataset, both models achieve the same performance for the *BG* and *TXT* classes, but S-U-NET scores higher for the *HL* and *GL* classes. For the UTP-110 dataset, L-U-NET performs better on the *BG*, *DEC*, and *FIL* classes, while S-U-NET has a slight advantage in the other classes, particularly in the *SDC* class.

#### 4.5.2. XAI Evaluation

In this work, we focus on assessing the attribution maps generated by each model. Table A1 and Table A2 compare different evaluation metrics: Infid, Sens_Max, CH, and ACS across various layers of the two U-NET models (L-U-NET and S-U-NET). In these two tables, each metric scores were aggregated using two techniques:

- **Average (A):** This represents the average metric score across all classes, including both foreground and background;
- **Foreground average (fA):** This represents the average metric score computed over the foreground classes only, excluding the background class.

Three key observations were deduced through Table A1 and Table A2:

**(i) Layer-wise analysis:** We observe that the best scores for Infid, CH, and ACS scores across all XAI methods correspond to the higher layers, especially FL, while the lowest scores tend to occur in the lower layers, particularly Dec4 for ACS, and Dec4 and Dec3 for Infid. For Sens_Max, we note similar trend for GradCAM, LRP, and G*I, with higher layers yielding better scores. However, for DeepLift, the lower layers outperform the higher ones.

**(ii) Foreground vs. background impact:** Analyzing the differences between *A* and fA provides insights into the impact of the background class on metric evaluations. For Infid, fA is consistently lower than *A*, indicating that the background contributes to higher Infid scores, mainly due to the inherent noise present in the background of HDIs. For Sens_Max, fA is lower than *A* in the lower layers, but gradually approaches *A* in the higher layers, eventually surpassing it for DeepLift. This suggests that DeepLift assigns greater relevance to deeper layers compared to other XAI methods. For ACS, the values do not exhibit a consistent trend, indicating that the influence of the background on this metric varies depending on the method and the layer being analyzed. This suggests that the background can have a misleading effect, as it is can bias the segmentation of the foreground classes. Hence, focusing only on the foreground classes helps mitigate the background impact on the XAI metrics.

**(iii) Model-wise comparison:** Comparing the performance of L-U-NET and S-U-NET across different explainability metrics provides insights into the models behavior. For Infid, L-U-NET achieves better performance in terms of *A* than S-U-NET. However, when considering fA, L-U-NET outperforms S-U-NET, particularly for GradCAM and DeepLift, while S-U-NET achieves better results for LRP and G*I. For Sens_Max, S-U-NET generally outperforms L-U-NET, except for DeepLift on the CB55 dataset, where L-U-NET achieves superior performance. For ACS, S-U-NET consistently achieves better performance than L-U-NET across all fA evaluations, highlighting its effectiveness in maintaining stable attributions in the foreground content.

#### 4.5.3. Qualitative Evaluation

In this section, we visually evaluate the generated heatmaps using the proposed workflow and analyze their alignment with the computed XAI metrics. We selected *TXT* and *TXTL* as target classes for the CB55 and UTP-110 datasets, respectively, as they represent textual features to ease the comparison process better between the two datasets. We additionally selected two XAI methods: Grad-CAM and LRP due to their fundamentally different underlying algorithms.

Figure A8 and Figure A9 and present sample attribution maps obtained at different decoder layers (*Dec4* to *FL*), from the CB55 and UTP-110 datasets, respectively. We observe differences in attribution quality and relevance distribution across the two U-NET architectures (L-U-NET and S-U-NET) and explainability methods (GradCAM vs. LRP), providing insights into the interpretability of the learned features at different layers.

In Figure A8 and Figure A9, the results of two XAI methods, GradCAM and LRP, are illustrated, with each row corresponding to one method. Each column represents the attribution map for a specific decoder layer, progressing from the deeper layers (*Dec4*) to the final layer (*FL*). The values of the Infid and ACS metrics are provided below each attribution map to quantitatively assess the quality of the attributions.

**(i) CB55:**

- **GradCAM:** For L-U-NET, we observe that *FL* has strong positive attributions on the *TXT* pixels, with minimal noise around borders. However, intermediate layers, particularly *Dec1* and *Dec3*, present intense noise, suggesting a negative effect caused by page borders on the model predictions. For S-U-NET, we also note similar positive attributions on the *TXT* pixels in *FL*, but with more distributed noise throughout the *BG* pixels. S-U-NET struggles more with noise in intermediate layers, particularly around the *GL* and borders, indicating challenges in distinguishing textual content from other document elements.
- **LRP:** For L-U-NET, we observe that the *TXT* pixels have positive attributions. However, there is only negative attributions within the text lines, especially in *Dec3* and *Dec4*, while the background shows little to no attribution. For S-U-NET, we observe that negative attributions are more pronounced within the *TXT* pixels compared to L-U-NET, creating gaps or holes, particularly in *Dec1* and *Dec2*. This suggests that S-U-NET struggles to consistently recognize the main text regions, starting from *Dec4* onward.

**(ii) UTP-110:**

- **GradCAM:** For L-U-NET, we observe a progressive refinement in attribution from *Dec4* to *FL*. In *Dec4*, positive attributions appear around the *TXTL* pixels and within the *BD* pixels, with negative attributions on the *BG* and *DEC* pixels. By *Dec3*, L-U-NET starts differentiating the *FIL* and *DEC* pixels. In *Dec1*, positive attributions focus on the *TXTL* pixels, while the *FIL* and *DEC* pixels have negative attributions. *FL* shows strong positive attributions for the *TXTL* pixels, while the *FIL* and *BG* have negative attributions, indicating the improved ability of L-U-NET to distinguish text from other document elements. For S-U-NET, we observe a similar trend to L-U-NET. In *Dec4*, positive attributions appear on the *TXTL* pixels, while the *DEC* pixels show strong negative attributions. By *Dec3*, negative attributions emerges between text lines, and in *Dec2*, the *SDC* pixels also receive negative attributions. *Dec1* further reinforces positive attributions on the *TXTL* pixels, while keeping negative attributions for the background. *FL* maintains strong positive attributions for the *TXTL* pixels and strong negative attributions for the *DEC* and *BG* pixels. Compared to the L-U-NET, S-U-NET struggles more with the *DEC* pixels, while L-U-NET finds the *FIL* pixels more challenging.
- **LRP:** Both models present similar behavior. In *Dec4*, both models have positive attributions on the *TXTL* regions, with S-U-NET showing concentrated negative attributions near the *FIL* and *DEC* pixels. As we move through *Dec3*, *Dec2*, and *Dec1*, negative attribution fades, with increasing focus on the *TXTL* pixels. For *FL*, both models strongly attribute the *TXTL* regions, with earlier negative attributions disappearing. The key difference is the stronger negative attributions of S-U-NET around *FIL* and *DEC* in *Dec4* compared to L-U-NET.

### 4.6. Human-Centric Alignment of XAI Metrics

To validate the plausibility of attributions, we introduce an evaluation protocol integrating a human assessment (*HA*) approach, conducted with a group of users and domain experts, as detailed in Section 3.5. The resulting human judgments are combined with the following three metrics: CH↑, PG↑, and ACS↑, computed across the five following layers: *Dec4* → *Dec1* and *FL*, on the CB55 and UTP-110 datasets.

#### 4.6.1. Human-Expert Assessment

To objectively assess the attribution maps, we complement the computed XAI scores with expert evaluations provided by domain specialists experienced in analyzing historical and administrative documents. For each dataset (CB55 and UTP-110), class, layer (*Dec4* → *Dec1* and *FL*), and XAI method (GradCAM, LRP, DeepLift, and G*I), experts inspected blended overlays and rated them for semantic plausibility and focus on class-relevant regions. We use a simple binary protocol with a stronger endorsement (✓✓ for clear, unambiguous focus; ✓ for acceptable focus; and × otherwise), resolving ties by majority vote. These *HA* marks, reported in Table A3 and Table A4, align with quantitative metrics (*PG*, *CH*, and *ACS*) and consistently favor higher layers—especially *FL*—where explanations concentrate on semantically meaningful regions.

#### 4.6.2. Metric Alignment

To quantify the agreement between human assessment (*HA*) and the computed XAI metrics, we calculate the mean squared error (*MSE*) between the *HA* score and each metric:

$${\mathrm{MSE}}_{m}={\left(m-{\mathrm{HA}}_{\mathrm{score}}\right)}^{2}$$

where *m* is the computed metric (*PG*, *CH*, or *ACS*).

All metrics are normalized to [0,1]. We map *HA* judgments to numeric targets as follows:

- ${\mathrm{HA}}_{\mathrm{score}}=1.0$ (✓✓ for clear, unambiguous focus);
- ${\mathrm{HA}}_{\mathrm{score}}=0.6$ (✓ for acceptable focus);
- ${\mathrm{HA}}_{\mathrm{score}}=0.0$ (× for bad focus).

Lower *MSE* indicates closer alignment with human judgment.

**Figure 13:**
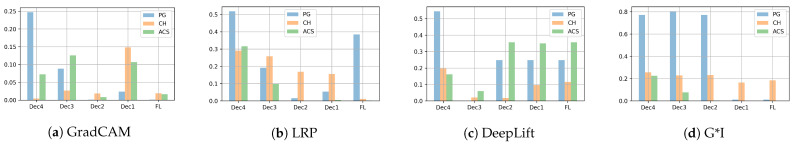
Comparative results on the CB55 dataset.

**(i) CB55:**Figure 13 reports the achieved average of *MSE* for *TXT*, *HL*, and *GL* across the four XAI methods using *PG*, *CH*, and *ACS*.

*ACS* aligns best for LRP and G*I. *CH* is strongest for DeepLift. *PG* excels for GradCAM in the last three layers (*Dec2*, *Dec1*, and *FL*). We attribute these discrepancies to dataset imbalance, especially the challenging *HL* class, which made human assessment of heatmaps harder and noisier.

**Figure 14:**
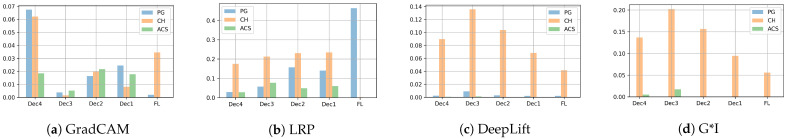
Comparative results on the UTP-110 dataset.

**(ii) UTP-110:** Figure 14 shows the achieved average of *MSE* for the decorator (*DEC*), filler (*FIL*), text line (*TXTL*), body (*BD*), small initial (*SInit*), and big initial (*BInit*) classes.

*ACS* consistently achieves the lowest *MSE* across methods, indicating that on a more balanced dataset *ACS* agrees well with human judgments of heatmap quality.

### 4.7. Saliency Overlay Analysis

**Figure A10:**
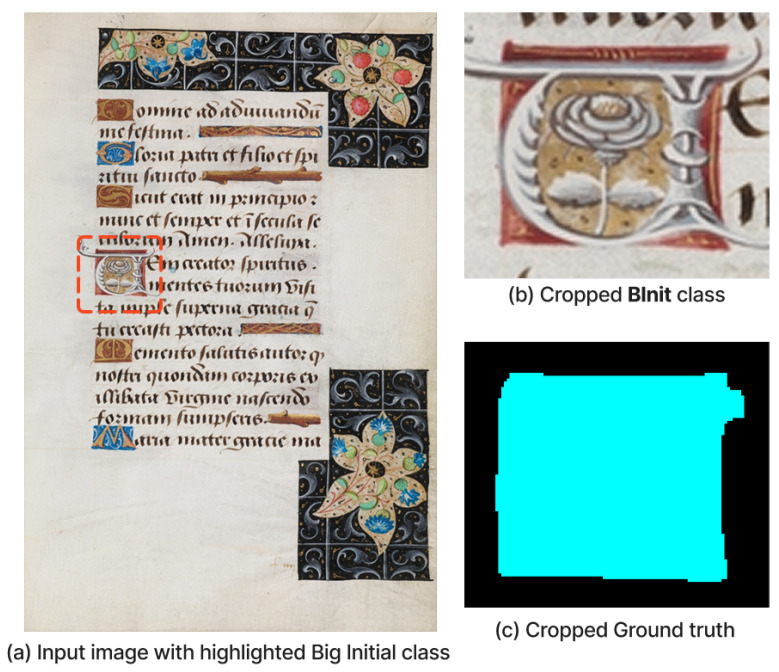
UTP-110 sample with cropped region of the *BInit * class (highlighted in red).

**Figure A11:**
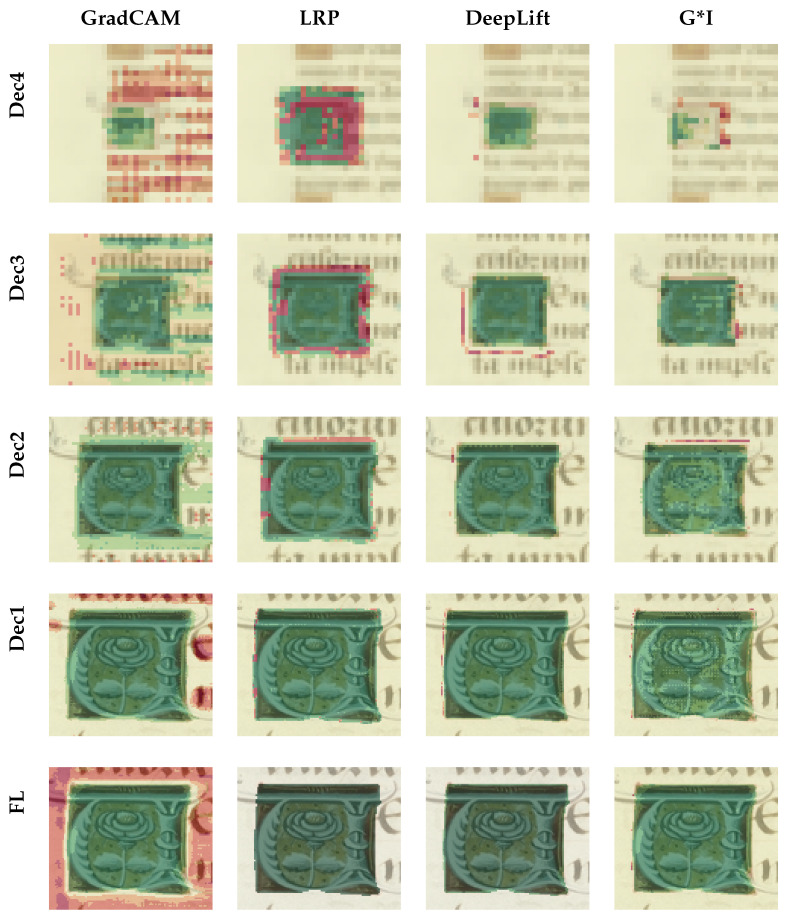
Evolution of the selected heatmap for the BInit class on the UTP-110 dataset using saliency overlay of the target input region.

To further investigate the interpretability of attribution maps, we select the *BInit* class from the UTP-110 dataset, which represents decorative glyphs with a large character on a colored background, as shown in Figure A10. We generate attribution maps using the four XAI methods across five decoder layers of S-U-NET and apply a saliency overlay restricted to the ground truth of the *BInit* region to visualize attribution alignment (see Figure A11).

Moving from deeper (*Dec4*) to shallower layers (*FL*) improves the quality of attribution maps, which become increasingly focused on the *BInit* region as noisy, off-target responses from early layers are refined.

- For GradCAM, heatmaps in *Dec4* and *Dec3* show strong interference from surrounding text, but from *Dec2* onward, attributions stabilize with positive activations concentrated within the *BInit* region.
- LRP initially produces mixed signals in *Dec4* but crucially captures the glyph’s internal “flower” symbol, and by *Dec2* converges toward dense positive attribution within the ground truth area. Notably, we apply a log normalization in Equation (23) to handle extremely high attribution values in *Dec1* while preserving patterns. Formally, given a attribution vector with *n* values
  $$Attr=(v_{1},v_{2},\ldots ,v_{n})$$
  each $v_{i}$ value is normalized as follows:
  (23) $$v_{i,\log}=\left\{\begin{array}{cc} \log_{10}\left(v_{i}\right) & \mathrm{if}\;v_{i}>1,\\ -\log_{10}\left(-v_{i}\right) & \mathrm{if}\;v_{i}<-1,\\ 0 & \mathrm{if}\;v_{i}\in \left[-1,1\right] \end{array}\right.$$
  where $v_{i}$ is the $i^{th}$ raw attribution value and $v_{i,\log}$ is its log-normalized form, preserving sign, compressing large magnitudes, and mapping values in [−1,1] to zero.
- DeepLift exhibits the most stable and consistent results, with positive attributions concentrating on the inner glyph from *Dec4* and becoming densely localized on the target by *Dec2*.
- In contrast, G*I fails to recognize the glyph in *Dec4*, producing scattered activations, but from *Dec3* onward, its maps became more structured and resembled DeepLift outputs.

### 4.8. Computational Cost

In this section, we present the computational costs of the two U-NET models on the CB55 and UTP-110 datasets, along with the costs of the applied attribution-based XAI methods and the computed XAI metrics.

Table 6 presents the computational costs for model architectures (inference time, learning time, and number of trainable parameters) of the two investigated U-NET variants on the two datasets. We note that L-U-NET, with 17,000 trainable parameters, took 5 h to train on the CB55 dataset and 2 h on the UTP-110 dataset. On the other side, S-U-NET, with over 31M trainable parameters, took 10 h to train on CB55 and 3 h on UTP-110. The resolution of images in the UTP-110 dataset is 640×960 (615 K pixels), which is smaller compared to the resolution of images in the CB55 dataset, which is 960×1344 (1.3M pixels). This results in shorter training and inference times for the UTP-110 dataset.

Table 7 presents the computational costs (time and memory) required to generate explanations in the form of attribution maps, as well as their evaluation on infidelity, sensitivity, ACS, and CH. It is important to point out that ACS and CH share the same values as they have the same time and resources complexities. For instance, ACS involves generating a single attribution map per input sample and target class using a selected XAI method. It then applies cheaper operations like masking to isolate the target region and separates the attribution map into positive and negative components before computing an F1-score. Similarly, CH relies on the same attribution map and aggregates the attribution values over the target region.

The Attribution column in Table 7 refers to the computational resources used to generate the attribution maps for all the images in the test set of each dataset and their all corresponding target classes. For example, using GradCam, the S-U-NET model took 101 s and 7.3 GB to run on the CB55 dataset, while only 85 s and 3.8 GB for the UTP-110 dataset. This validates that image resolution significantly impacts resource consumption. For CB55, with 4 target classes, generating an attribution map for a single image takes approximately 1.26 s, whereas for UTP, with 7 target classes, it takes only 0.60 s.

From a computational perspective, the dominant cost for both metrics is mainly due to attribution map generation as this process typically involves a full forward pass through the whole model and an addition backward pass or, in the case of methods such as Grad-CAM, a partial backward pass. These steps are considerably more resource-intensive than the subsequent operations of masking or aggregation, which contribute minimally to the overall time and memory requirements.

When comparing the XAI methods, LRP tends to be the slowest, while Grad-CAM is the fastest. DeepLIFT, on the other hand, has the highest memory consumption. On extreme cases, S-U-NET exceeded the 32 GB memory limit of our hardware, forcing us to switch to the CPU, which significantly increased the computation time. GradCAM and G*I have similar computation times and memory usage, primarily due to their comparable computational complexities. Both methods rely on calculating gradients with respect to the model output and using backpropagation to compute these gradients.

Each of the computed XAI metrics uses different algorithms and varies in resource requirements. Among them, Sens_Max is the most resource-intensive. In terms of computation time, Infid has the highest computation time in most cases. ACS and CH have the lowest resource consumption among the computed metrics, as they only rely on a single attribution map computation. Finally among all the used models, S-U-NET on CB55 consumed the most resources, while L-U-NET on UTP-110 consumed the least.

### 4.9. Synthesis

Based on the achieved results, a concise synthesis highlighting our key findings and observations is proposed in this section.

From an interpretability standpoint, DNNs continue to pose many challenges, even with the integration of XAI methods. For instance, attribution maps, at a superficial level, may appear to validate model decisions when observing only the final segmentation layer, but this perspective can be misleading, as earlier layers often reveal hidden biases or non-obvious decision pathways within the model. For example, on the CB55 dataset, the S-U-NET architecture exhibits a noticeable gap in attributing textual content using the LRP method (see Figure A8). Negative attributions remain ambiguous until layer *Dec4*, where underlying factors begin to emerge. Similarly, for L-U-NET on the same dataset, the attribution of *FL* suggests strong alignment with the *TXT* pixels while attributing all other regions negatively. However, by examining preceding layers, we observe early signs of negative attribution in the *GL* pixels and along the page borders. These patterns reinforce the importance of a layer-wise interpretability approach, as used in our proposed workflow, rather than relying solely on the final output layer.

Our interpretation also highlights that explanation improves consistently from deeper decoder layers *Dec4* to *FL*, as confirmed by both the *Infid* and *ACS* metrics. This reflects the decoder’s role in gradually fusing abstract features with high-resolution details from the skip connectors in U-NET.

We also report that S-U-NET produces more plausible and robust explanations compared to L-U-NET qualitatively judging on heatmaps, and on both high values of *ACS* and lower *Sens_Max* values. On the other hand, L-U-NET produces more faithful explanations with low *Infid* values. Thus, lightweight models contribute to more predictable explanation maps matching the model prediction.

Based on the quality of the generated attributions, we draw the following conclusions regarding the attribution methods:

- GradCAM is efficient in terms of memory consumption and capable of highlighting negative attributions through gradients in the deeper layers. However, its reliability diminishes in lower or intermediate layers due to the gradient vanishing issues.
- LRP demands greater computational resources but produces more consistent and informative attribution maps. Its ability to detect deeper anomalies stems from its adherence to the conservation of information principle.

When comparing the two datasets, UTP-110 consistently produces coherent, high-quality heatmaps. In this balanced dataset with distinct document components, positive attributions are strongly concentrated on target classes, while negative attributions are distributed over non-target regions, which leads to higher *ACS* values and clearer interpretability. By contrast, the CB55 dataset is more challenging: heatmaps are harder to interpret, particularly for the *HL* class, which separates text paragraphs, and the *GL* class, which is frequently confused with the *TXT* class.

We conclude that the dataset composition significantly affects the model predictions as well as the interpretability of attribution maps. Selecting well-separated document components, each with distinct shapes and features, enables the model to:

1. Build more robust feature representations;
2. Assist the segmentation head in producing clearer segmentation boundaries;
3. Enhance compatibility with a wide range of XAI methods for both visual and quantitative heatmap evaluations.

While increasing the number of target classes does not inherently create new neural pathways in the network, precise ground truth annotations and well-defined class separations substantially contribute to improved model predictions and more interpretable attribution maps.

## 5. Generalization

**Figure 15:**
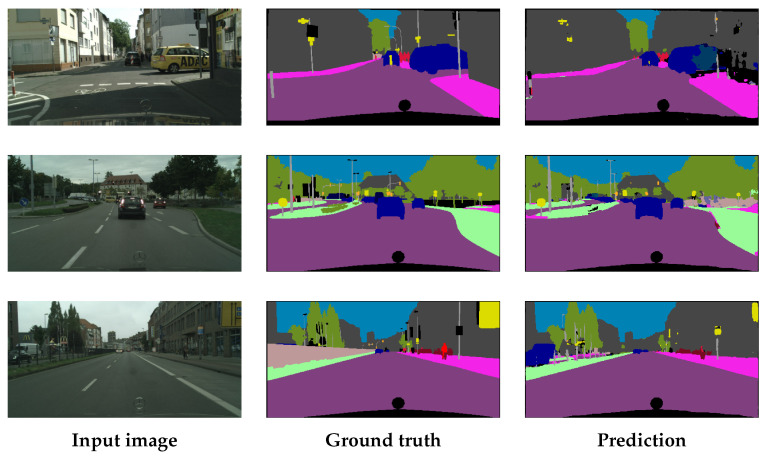
Cityscapes examples showing input images, corresponding ground truth, and predictions.

This section evaluates the domain transferability of the proposed workflow and validates its generalization by extending our experiments beyond HDI. Specifically, we conduct additional experiments on Cityscapes, one of the most widely used and complex benchmarks for urban scene segmentation [32]. Cityscapes includes 5000 finely annotated and 20,000 coarsely annotated street-view photos from 50 European cities for interpreting urban scenes (see Figure 15). Captured using a car-mounted camera, Cityscapes gives pixel-level labels in a 20-class arrangement (e.g., road, building, automobile, pedestrian) and is commonly used to train and evaluate semantic segmentation for autonomous driving [53,54].

Due to the large number of samples of Cityscapes compared to CB55 and UTP-110 datasets, only *CH*, *PG*, and *ACS* are computed. Although L-U-NET suffers from underfitting due to its limited number of learnable parameters, L-U-NET and S-U-NET are both trained for 200 epochs. For the Cityscapes dataset, the same XAI methods and selected layers are used as for the two HDI datasets. We also introduce a user study (see Table A5), integrating human-in-the-loop feedback, where several users manually assessed the generated heatmaps and assigned scores.

These experiments focus on evaluating how well each metric reflects an XAI method ability to explain a target class in the input image relative to human interpretation. The end goal is to quantify inter-rater agreement among users in relation to one or more metrics.

When evaluating performance on Cityscapes, we observe that the model predicts classes missing from the ground truth. This usually happens in dense, multiclass urban datasets like Cityscapes, where one image can contain many different object types, unlike HDI datasets with only a few categories. Therefore, these issues should be taken into consideration when evaluating XAI methods: *(i)* dataset artifacts that can mislead explanations, *(ii)* model quality (under/overfitting) that yields erroneous predictions, and *(iii)* the capability of the explanation method itself to extract meaningful insights from the model.

To address these issues, we include two different approaches to compute the metrics. The first approach relies on computing the average metrics on the predicted target classes, which is the standard in the state-of-the-art. The second approach relies on using only the intersection between the predicted classes and the classes that are annotated in the ground truth. The motivation behind the second approach is to mitigate the impact of incorrect classes in the predicted output, which could otherwise distort the explainability measure.

**Figure 16:**
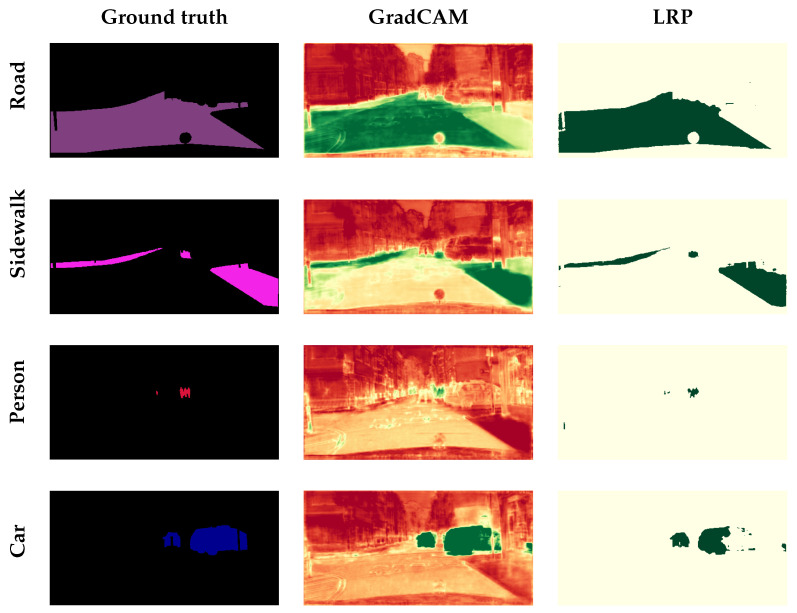
Comparison of two XAI methods (GradCAM and LRP) across four different target classes: road, sidewalk, person, and car.

In Figure 16, we provide additional qualitative results using targeted heatmaps for different classes in Cityscapes. These heatmaps are generated with GradCAM and LRP applied to the last layer of the S-U-NET architecture.

The quantitative results are reported in Table A5, evaluated with different targets across the same five U-NET layers used for both the CB55 and UTP-110 datasets.

**Figure 17:**
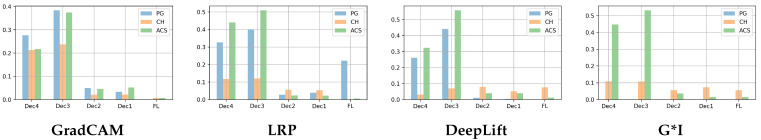
Comparative results on Cityscapes.

Similar to the comparative study in Section 4.6.1, we conducted additional experiments to evaluate the generalization of SegClarity on the Cityscapes dataset (see Figure 17). Across all attribution methods, *ACS* achieves the best performance at layer *Dec2*, and performs comparably to *PG* at the final layer (*FL*). However, for earlier layers (*Dec4* and *Dec3*), both *CH* and *ACS* scored poorly. This is largely due to the low-resolution feature maps at those stages (32×64 and 64×128), where upsampling introduces significant distortion. As a result, nearly all *Dec4* and *Dec3* heatmaps are rated as poor by human evaluators. Therefore, for Cityscapes, higher-resolution layers (*Dec2*, *Dec1*, and *FL*) provide more reliable and interpretable attribution maps.

**Figure 18:**
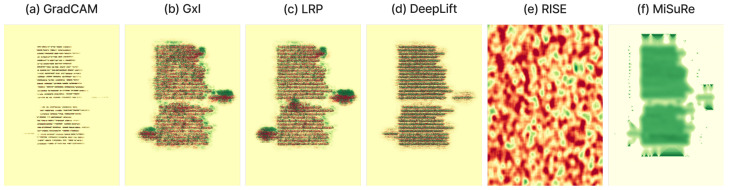
Qualitative comparison of attribution maps for the main text class on the CB55 historical document dataset using six attribution methods.

**Figure 19:**
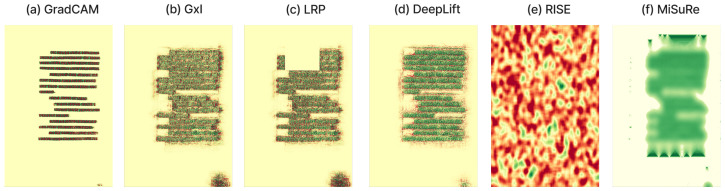
Qualitative comparison of attribution maps for the main text class on the UTP110 historical document dataset using six attribution methods.

**Figure 20:**
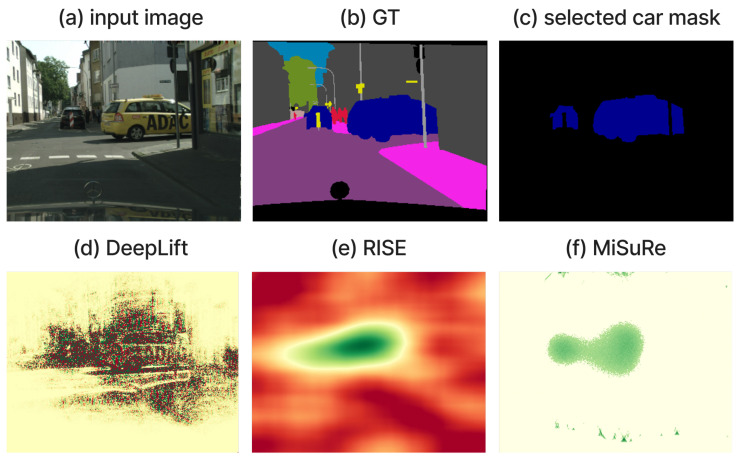
Qualitative evaluation of cross-domain evaluation on the Cityscapes dataset for the car class. An input image (**a**) of ground truth mask (**b**) containing a car instance (**c**) is assessed using a decomposition-based method, DeepLift (**d**), and two perturbation methods, (**e**) RISE and (**f**) MiSuRe.

Beside layer-wise method comparison, we also include an additional study on the input layer explanation. The qualitative results presented in Figure 18, Figure 19 and Figure 20 highlight the strong domain dependency of explainability methods in segmentation tasks. For historical document datasets (CB55 and UTP-110), gradient and decomposition-based methods such as Grad-CAM, Gradient × Input, LRP, and DeepLIFT produced coherent and semantically meaningful attributions closely aligned with textual regions, whereas perturbation-based methods like RISE and MiSuRe failed to capture the fine-grained layout structure or produced overly diffuse activations. In contrast, on the Cityscapes dataset, all methods—including RISE and MiSuRe—generated stable and contextually relevant explanations, demonstrating their suitability for natural image domains. These observations confirm that XAI methods, although categorized as being agnostic, must be adapted to the visual characteristics of the target domain, as document image analysis requires finer structural sensitivity and dedicated attribution strategies to achieve reliable interpretability. We can also confirm this interpretation by computing three quantitative metrics (CH, PG, and ACS) for the input-layer attributions generated on the three datasets. The results are summarized in Table 8. For the CB55 dataset, the best performance is achieved by *Grad-CAM* on ACS and PG, while *MiSuRe* yields the highest CH score. In the UTP-110 dataset, *DeepLIFT* obtains the best ACS and PG values, and *MiSuRe* again leads in CH. Finally, for the Cityscapes dataset, *MiSuRe* achieves the highest ACS, whereas *Grad-CAM* and *LRP* exhibit the strongest results for PG and CH, respectively.

## 6. Discussion

In this article, we present an explainability workflow tailored for the document analysis community and XAI practitioners working with semantic segmentation applications. Our workflow is based on attribution maps, providing a comprehensive analysis of model interpretability both visually and through a set of quantitative measures to enhance the evaluation of DNNs. By leveraging various categories of XAI methods, we demonstrated the extent of information that can be extracted from a trained DNN and how these insights can be used to interpret and compare multiple DNNs using the proposed contributions.

Our findings highlight the importance of selecting appropriate XAI methods and metrics for semantic segmentation tasks. Unlike classification, where attribution maps often align with class activation maps, segmentation poses unique challenges:

- They are inherently difficult to interpret, as attribution maps do not always resemble segmentation masks;
- Applying and adapting the existing XAI methods and measures to rich datasets with pixel-wise annotations presents significant computational challenges.

In our previous work [21], we addressed these issues by introducing a framework for interpreting layout analysis models, employing three XAI methods and two evaluation metrics. This work extends this framework by investigating the LRP method and providing a broader set of metrics for evaluation. Furthermore, we demonstrated that a robust interpretability assessment should extend beyond the final output layer, as commonly used in the literature, and instead consider multiple levels of the network. Furthermore, we introduced a targeted perturbation technique for the infidelity metric and the ACS, a novel metric that aligns with human reasoning, providing mathematical justification for its formulation and showcasing its effectiveness in this context.

Throughout this work, we clearly observe the importance of carefully handling model explanations, as they provide valuable insights into model behavior. For instance, known perturbation-based methods, such as RISE, are dependent on the task and the quality of input data. We demonstrated in the end of the generalization section that document layout analysis is different from other segmentation domains due to its rich nature of textual pixels and its highly correlated components. MiSuRe, on the other hand, kept scoring stable results regardless of the task. We argue that MiSuRe is, at its core, suitable for a variably of data, because it compromises with an optimization phase to be more selective in the choice of saliency map.

Visually inspecting the model explanations without prior knowledge or suitable pre-processing can lead to significant misinterpretations. For instance, the LRP method outputs large values and creates a large gap in the distribution of attribution map values. Regarding the U-NET architecture, higher decoder layers tend to be more interpretable than lower layers closer to the bottleneck. This is due to the fact that when moving up in the decoder, U-NET is able to extract finer details from the input and produce higher-resolution output. This observation is supported by the computed values of the ACS and Infid metrics. However, it is reversed for the DeepLift method when using Sens_Max. This highlights the importance of carefully selecting the appropriate XAI method based on the specific task and dataset.

As further evidence of the alignment between our metric and human perception, we conducted a human evaluation phase in which participants were asked to judge the interpretability of the generated heatmaps using the proposed workflow. Among the computed metrics, *PG*, *CH*, and *ACS*, *ACS* consistently showed the strongest agreement with human assessments. Although *ACS* achieved the best overall performance, it exhibited a bias toward balanced datasets, underscoring the importance of data quality and class distribution in interpretability. In addition to human evaluation, the proposed workflow was applied to a dataset beyond the field of HDI analysis. The results remained consistent, demonstrating that SegClarity can be adapted to a wide range of segmentation tasks.

The choice of layers to interpret impacts the quality of attribution. In our case, we chose the input layer, like the majority of works on XAI, as well as intermediate layers in the network. Although we only consider the decoder part of the network, other components can provide additional insight to studying such models:

- The encoder blocks as we progress through the network in a downsampling manner as well as the choice of encoder type to employ;
- The skip connections where an interesting study can be included by measuring the impact of each skip connection on the decoder block.

The results also suggest a strong correlation between model performance and interpretability, as highlighted in prior studies [15,55,56]. This work could also be more developed if we explore more explainability methods. For instance, the randomized input sampling for explanation (RISE) [48] method relies on image ablation and impact assessment to generate attribution maps where we can use our perturbation method alongside it to produce meaningful perturbations for segmentation tasks and reduce time complexity of RISE. Another area to explore is the LRP variants, such as LRP-γ, LRP-αβ, and deep Taylor decomposition (DTD). Regarding the Sens_Max metric, the perturbation radius is still not studied due to the high complexity and computational costs for segmentation tasks; for that, we strongly advise practitioners to look for more optimized ways for the sensitivity measurements, especially for costly methods, such as LRP and DeepLift.

## 7. Conclusions

In this work, we present a comprehensive workflow, called SegClarity, that integrates DNNs and XAI methods to advance the task of analyzing the layouts of HDIs. By addressing multiple challenges, such as the scarcity of annotated data and the complexity of document layouts, we emphasize the importance of optimized architectures and interpretable models. Our work highlights the role of XAI in enhancing the transparency and reliability of DNNs. By providing a variety of attribution- and perturbation-based XAI methods (GradCAM, DeepLift, LRP, G*I, RISE and MiSuRe), we provide valuable insights into model behavior and decision-making processes. We also propose a custom perturbation method based on semantic annotations and tailored to pixel-wise prediction tasks. This method generates meaningful perturbations which, when combined with an evaluation metric like Infid, can assess whether the highlighted regions in an explanation effectively influence the model’s predictions. The introduction of a novel XAI metric, called ACS, further strengthens the evaluation of explainability for pixel-wise segmentation, providing a robust metric for aligning model attributions with the complex layouts of HDIs. In addition to supporting existing evaluation methods, ACS offers an additional perspective into the assessment of XAI methods in terms of explanation plausibility. Through quantitative and qualitative evaluation using SegClarity, we demonstrated that layer-wise analysis reveals deeper understanding of model behavior and enables a more effective assessment from an interpretability and explainability perspective.

A key contribution of this work is the domain independence of SegClarity and the effect of generalization on the explanation. By applying SegClarity to the Cityscapes dataset, we demonstrate its versatility in the complex task of urban scene segmentation. This generalization confirms that the layer-wise analysis, visualization tools, and *ACS* metric provide consistent and meaningful insights across fundamentally different applications: from detecting textual glosses in medieval manuscripts to segmenting roads, vehicles, and pedestrians in modern street scenes. These results establish SegClarity as a versatile, domain-agnostic framework for evaluating and interpreting pixel-wise prediction models. Additionally, we demonstrated that traditional perturbation methods are domain-dependent, especially in the case of RISE, and require careful consideration their integration. These findings suggest that the document analysis domain requires specialized XAI adaptations, as not all conventional approaches are directly applicable to such structured visual data.

This work contributes to the growing field of explainability in document layout analysis, fostering trust and interpretability in DNN-based systems. Our findings serve as a benchmark for future research efforts, driving the development of advanced tools for segmenting HDIs and facilitating the preservation and dissemination of cultural heritage. Through the integration of efficient architectures and XAI methods, we aim to bridge the gap between technological innovation and practical application in the analysis of HDIs.

As future work, we plan to leverage SegClarity to actively impact model behavior during training, ensuring that the learning process is guided toward improved performance and enhanced generalization. By incorporating attribution-based insights during training, we aim to refine feature learning and reduce potential biases, leading to more robust document layout segmentation. Furthermore, we intend to extend our work by building upon the research of Achtibat et al. [57] on concept relevance propagation (CRP). Specifically, we intend to adapt and integrate CRP into our workflow, similar to the work introduced by Dreyer et al. [50]. This extension will enable us to explore concept-based attribution in HDI segmentation, further aligning model attributions with human-interpretable document structures and enhancing the overall explainability of DNNs dedicated to layout analysis. Although our work centers on document analysis as a case study, the proposed workflow and insights open promising perspectives for broader applications. In particular, they can be extended to other pixel-wise segmentation tasks, such as those in medical imaging [58,59,60] and beyond.

## Figures and Tables

**Table 1:** Comparison of the six XAI methods evaluated in this study. The methods are grouped into three categories: gradient-based (*Grad-CAM*, *Gradient × Input*), decomposition-based (*LRP*, *DeepLIFT*), and perturbation-based (*RISE*, *MiSuRe*). Each method is characterized by its underlying principle and whether it supports input-level or intermediate-layer attributions using the (✓) symbol, otherwise we indicate the opposite using the (×) symbol.

Method	Type	Input Layer	Intermediate Layer
Grad-CAM	Gradient-based	✓	✓
G × I	Gradient-based	✓	✓
LRP	Decomposition-based	✓	✓
DeepLIFT	Decomposition-based	✓	✓
RISE	Perturbation-based	✓	×
MiSuRe	Perturbation-based	✓	×

**Table 2:** Spatial dimensions (height × width) of the decoder layers in the CB55 and UTP-110 datasets.

Layer	Spatial Dimensions	
	CB55	UTP-110
*Dec4*	168×120	120×80
*Dec3*	336×240	240×160
*Dec2*	672×480	480×320
*Dec1*	1344×960	960×640
*FL*	1344×960	960×640

**Table 3:** Mask size sensitivity analysis for the infidelity perturbation metric. Each value represents the mean ± standard deviation over 100 iterations and 50 random patches. The fifth row with bold values indicate the best configuration for our perturbation experiment.

Patch Size	Confidence Drop	IoU Drop	DICE Drop	Pixel Change Ratio
2 × 2	0.0000 ± 0.0001	0.0036 ± 0.0084	0.0028 ± 0.0085	0.0006 ± 0.0008
4 × 4	0.0000 ± 0.0001	0.0077 ± 0.0136	0.0064 ± 0.0144	0.0013 ± 0.0009
8 × 8	0.0001 ± 0.0001	0.0138 ± 0.0138	0.0107 ± 0.0134	0.0023 ± 0.0012
16 × 16	0.0001 ± 0.0001	0.0284 ± 0.0261	0.0240 ± 0.0290	0.0043 ± 0.0017
32 × 32	**0.0002 ± 0.0002**	**0.0540 ± 0.0317**	**0.0445 ± 0.0345**	**0.0088 ± 0.0022**
64 × 64	0.0001 ± 0.0003	0.1095 ± 0.0364	0.0944 ± 0.0436	0.0174 ± 0.0033

**Table 4:** Experimental setup for perturbation-based methods (RISE and MiSuRe) across document datasets (CB55, UTP-110) and Cityscapes. Parameter values were empirically selected to maximize method performance and ensure stable convergence.

Parameter	CB55/UTP-110	Cityscapes
Metric	DICE	DICE
Patch size (w,h)	32×32	8×8
Number of masks	500	500
Batch size	1	8
Probability threshold $\left(p_{th}\right)$	0.5	0.5
Total variation weight $\left(\gamma_{tv}\right)$	0.001	0.001
Score mode	dice	dice
Sparsity weight $\left(\lambda_{s}\right)$	0.01	0.01
Learning rate	0.1	0.1
Foreground weight $\left(\alpha_{fg}\right)$	2	2
Background weight $\left(\alpha_{bg}\right)$	1	1
Temperature (τ)	0.9	0.9
Mask size	(960,1344) (CB55), (640,960) (UTP)	(256,512)

**Table 5:** Comparison of performance using standard semantic segmentation metrics (accuracy, precision, recall, F1-score, and IoU) between the L-U-NET and S-U-NET models on the CB55 and UTP-110 datasets. The bold values indicate the best recorded metrics for each model on each dataset.

Dataset	Model	Accuracy	Precision	Recall	F1-Score	IoU
CB55	L-U-NET	0.914	**0.926**	0.914	0.920	0.855
	S-U-NET	**0.935**	0.925	**0.935**	**0.929**	**0.870**
UTP-110	L-U-NET	**0.951**	0.912	**0.951**	0.930	0.870
	S-U-NET	0.946	**0.935**	0.946	**0.940**	**0.889**

**Table 6:** Computational cost comparison of the L-U-NET and S-U-NET models across the CB55 and UTP-110 datasets.

Model	#Params	Dataset	Inference Time	Training Time
L-U-NET	17K	CB55	4.82 s	5 h
		UTP-110	3.33 s	2 h
S-U-NET	31M	CB55	4.81 s	10 h
		UTP-110	3.53 s	3 h

**Table 7:** Computational costs required to generate explanations in the form of attribution maps using four different XAI-based methods, as well as their evaluation after applying *L-U-NET* and *S-U-NET* on the images of the test set of the CB55 and UTP-110 datasets. The cells highlighted in red represent the largest values among all the method.

Dataset	Model	Method	Attribution Maps		Infid		Sens_Max		CH and ACS	
			Time (s)	Memory (GB)	Time (s)	Memory (GB)	Time (s)	Memory (GB)	Time (s)	Memory (GB)
CB55	L-U-NET	GradCAM	22	2.2	480	5.9	300	7.5	35	3.2
		LRP	51	5.5	540	6.5	600	19	104	6.6
		DeepLift	36	11.4	480	8	480	26.5	50	8.8
		G*I	22	2.2	480	5.6	300	7.5	36	3.2
	S-U-NET	GradCAM	101	7.3	2040	21.5	1200	27.3	129	9.7
		LRP	235	23	2400	27.6	2760	29.9	285	24
		DeepLift	~5400	36.6	~50,400	32	~56,300	98.6	~5400	30
		G*I	100	7.3	2040	21.5	1260	27.3	159	9.7
UTP-110	L-U-NET	GradCAM	20	1.2	900	3.5	240	4.3	33	2.2
		LRP	43	3	1020	3.8	540	9.9	57	3.8
		DeepLift	32	3.5	900	4.4	420	13.3	44	5
		G*I	20	1.2	900	3.5	240	4.4	32	2.2
	S-U-NET	GradCAM	85	3.8	~3600	11	~1080	13.8	157	5.4
		LRP	196	11	~3600	13.9	~2280	16.5	266	12.1
		DeepLift	159	23.3	~93,600	15.9	~48,500	47.7	172	15
		G*I	85	3.8	~3600	11	~1080	13.8	160	5.4

**Table 8:** Quantitative comparison of XAI methods across datasets (CB55, UTP-110, and Cityscapes) for input-layer explanations. Reported metrics include content heatmap (CH), pixel grouping (PG), and Attribution Concordance Score (ACS). The ACS values correspond to the Otsu-thresholded variant. The ↑ symbols indicate that higher values are better. The ↑ indicate that the highest values are the better. The green cells indicate that the current method scored the best values in that dataset.

Dataset	Method	CH ↑	PG ↑	ACS ↑
CB55 (Text)	DeepLIFT	0.415	1.000	0.630
CB55 (Text)	Grad-CAM	0.569	1.000	0.738
CB55 (Text)	Input × Gradient	0.554	0.000	0.391
CB55 (Text)	LRP	0.637	0.000	0.025
CB55 (Text)	MiSuRe	0.692	0.000	0.544
CB55 (Text)	RISE	0.558	0.000	0.181
UTP-110 (Text)	DeepLIFT	0.451	1.000	0.832
UTP-110 (Text)	Grad-CAM	0.553	1.000	0.735
UTP-110 (Text)	Input × Gradient	0.421	0.000	0.588
UTP-110 (Text)	LRP	0.306	0.000	0.647
UTP-110 (Text)	MiSuRe	0.807	0.000	0.601
UTP-110 (Text)	RISE	0.448	1.000	0.249
Cityscapes (Car)	DeepLIFT	0.474	0.000	0.627
Cityscapes (Car)	Grad-CAM	0.661	1.000	0.793
Cityscapes (Car)	Input × Gradient	0.637	0.000	0.748
Cityscapes (Car)	LRP	0.601	1.000	0.678
Cityscapes (Car)	MiSuRe	0.480	0.000	0.910
Cityscapes (Car)	RISE	0.557	1.000	0.721

## Data Availability

The original contributions presented in this study are included in the article. Further inquiries can be directed to the corresponding author.

## Appendix A

### Appendix A.1. Attribution Generation

#### Appendix A.1.1. Segmentation Adaptation

XAI attribution methods are predominantly designed for image classification, where models generate a single class-score vector per image. By contrast, semantic segmentation models generate dense predictions as a tensor of size [B,C,H,W], where *B*, *C*, *H*, and *W* correspond to the batch size, number of prediction classes, and the height and width of the segmentation map, respectively, with class probabilities or logits assigned independently to each pixel. Due to this mismatch in output format, classification-based XAI methods cannot be directly applied. To bridge this gap, we adapt the segmentation model output into a classification-compatible form that can be seamlessly integrated with existing XAI techniques. The core idea is to convert the per-pixel predictions into a single scalar score per class, thereby emulating the structure of a classification output. Through this adaptation, we can directly apply well-established attribution methods, eliminating the need for re-implementation.

Algorithm A1, adapted from the Captum XAI library [61] (https://captum.ai/tutorials/Segmentation_Interpret, accessed on 8 November 2025), formalizes the adaptation of segmentation outputs for integration with XAI techniques. First, we compute the model output *Y* and determine the original per-pixel argmax prediction, denoted pred. A one-hot mask *M* is then constructed to fix the predicted class at each pixel. By multiplying this mask with the original output, we retain only the logits corresponding to the predicted class for each pixel. Finally, these logits are summed over the spatial dimensions (H,W) to yield a scalar score for each class, producing a vector $s\in R^{B\times C}$ that mirrors a classification output. This adaptation presents several advantages. First, it preserves stability during attribution: by linking the mask to the original predictions, the target class remains consistent even when XAI techniques require input perturbations. Second, it enables the direct re-utilization of robust and optimized XAI techniques initially adopted for classification tasks, ensuring that trials are both efficient and comparable.

**Algorithm A1 Segmentation Adaptation**
Input: *f* (segmentation model), *X* (input batch), *C* (number of classes) Output: s (class-wise scalars) 1. Y←f(X) *segmentation logits, $Y\in R^{B\times C\times H\times W}$* 2: $\mathrm{pred}\leftarrow \arg\max_{c}Y$ *predicted class index per pixel, shape [B,1,H,W]* 3: Construct $M\in {\left\{0,1\right\}}^{B\times C\times H\times W}$ with M[b,c,h,w]=1 if c=pred[b,1,h,w], else 0 4: Z←Y⊙M *retain only logits of the predicted class at each pixel* 5: $s\leftarrow \sum_{h,w}Z$ *aggregate over spatial dimensions; $s\in R^{B\times C}$* 6: **return** s

#### Appendix A.1.2. Attribution Computation

Our workflow starts by generating fine-grained attribution maps using an attribution-based XAI method, an input vector *x*, a layer name *l*, and target class to explain *t*.

Given a segmentation model *f*, composed of an ordered set of layers, each layer is a parametric mapping $l_{i}:X_{i-1}\to X_{i}$, where $X_{i-1}$ and $X_{i}$ denote the input and output spaces of the layer, respectively. The overall model is then expressed as the following composition:

$$f\left(x\right)=l_{n}\circ l_{n-1}\circ \ldots \circ l_{1}\left(x\right)$$

To ensure precise referencing, each layer is assigned a unique identifier or name, allowing the network to be represented as a collection of named modules. Accordingly, *f* is defined as follows:

$$f=\{\;\left(\mathrm{name}\left(l_{1}\right),l_{1}\right),\left(\mathrm{name}\left(l_{2}\right),l_{2}\right),\ldots ,\left(\mathrm{name}\left(l_{n}\right),l_{n}\right)\;\}$$

Given a particular name, we denote by l∈f the unique layer corresponding to that identifier. For example, in a U-NET segmentation model, we may refer to a decoder block by the name Dec2, so that retrieving l=Dec2 gives access to its intermediate feature maps.

An attribution map is a vector of numbers that represents the method interpretation of the model behavior given the target class to interpret. Based on the DNN output and gradients, our workflow calculates the contributions for the attribution maps, ensuring that the structure of the generated maps aligns with the corresponding layer shape.

First, to compute the raw prediction of a model for a given input image *x*, we perform a forward pass through the network *f*. This process yields a dense output tensor, denoted as out, which is defined as follows:

$$out=f\left(x\right)\in R^{C\times H\times W}$$

This dense, pixel-wise output is not directly suitable for attribution XAI-based methods designed for classification tasks. Therefore, if we apply the adapter function (as detailed in Algorithm A1) to aggregate these scores, we will obtain a class-wise score vector *s*, defined as follows:

$$s=wrap\left(f,x,t\right)\in R^{C}$$

where wrap is the adapter function that adapts the segmentation output into a classification-like representation. Given the model *f*, input *x*, and target class *t*, it aggregates the dense prediction tensor into a vector *s* of length *C*.

We then define Attr, a generic attribution function, as follows:

(A1) Attr=g(f,s,ℓ),

where

- *f* denotes the model, typically a DNN for segmentation or another prediction task;
- *s* is the selected output, a scalar quantity derived from the model predictions (e.g., the logit for a specific class at a pixel, or a guided score pooled over a region of interest);
- *ℓ* is the selected internal layer (input, intermediate, or output) whose contribution we aim to analyze.

This process integrates the generation of the map $Attr\in R^{H\times W}$ with the DNN architecture, and afterwards it is transformed into a visualization that is both informative and representative of the DNN internal workings. The attribution map generally includes *favorable (positive) attributions* linked to features that support the DNN prediction, *unfavorable (negative) attributions* indicating deviation from the DNN judgment, and neutral attributions with limited impact.

To generate attribution maps, four state-of-the-art attribution-based XAI methods are used in our work, specifically two gradient-based techniques (Grad-CAM and G*I) and two decomposition-based approaches (LRP and DeepLIFT). Perturbation-based methods are excluded from our analysis, as we aim to introduce perturbation metrics in our assessment. Each method provides unique characteristics that contribute to achieving a more comprehensive understanding of model behavior. Grad-CAM operates through gradient propagation, while LRP relies on relevance attribution with the mathematical property of information conservation. DeepLIFT combines gradient-based analysis with reference-based comparisons, and G*I serves as a baseline method driven by neuron activation.

In the process of generating attribution maps, we have customized Grad-CAM and LRP by adapting their implementations to meet our specific application needs.

For GradCAM, it is common practice to apply a ReLU function to the generated heatmap, thereby focusing solely on the features that positively influence the prediction of the target class (https://christophm.github.io/interpretable-ml-book/pixel-attribution.html, accessed on 8 November 2025). However, to capture a more comprehensive understanding of the model behavior, including both positive and negative attributions, we have modified the Grad-CAM implementation by omitting the ReLU operation.

For LRP, it is imperative to tailor the attribution map generation function by assigning propagation rules that correspond to the specific operations of each layer. This customization ensures that the relevance scores are accurately propagated through the network. For instance, convolutional layers, which apply filters to extract spatial features, should utilize the ϵ-rule to maintain numerical stability during relevance propagation. Activation functions, such as ReLU, which introduce non-linearity by zeroing out negative inputs, are best suited to the identity rule, allowing relevance to pass through unchanged. Normalization layers, such as InstanceNorm, which standardize the input to have a specific mean and variance, should also employ the ϵ-rule to ensure consistent relevance distribution. A comprehensive research work focusing on selecting the appropriate propagation rules for various layer types in DNNs was introduced by Montavon et al. [62].

The generated attribution maps are subjected to additional processing to improve their visuals. This involves separating positive and negative characteristics and overlaying them on input image to generate a combined heatmap. In Figure 4, we display positive and negative attributions in distinct columns, providing valuable insights into the significance of features for model predictions. To further elucidate the model’s prospective, we present a superimposed blended heatmap overlaid on the input image. This visualization integrates of the highest positive attribution and negative attribution values. These regions are overlaid with an opacity of 0.5 to enhance visual interpretability. In this example, in Figure 4, a 70% threshold for both positive and negative attribution values and an opacity level of 0.5 were selected based on empirical observations. These parameters are adjustable and can be tailored according to the specific method employed and the characteristics of the input image. Using both separate and blended visualizations helps to better understand model behavior and supports its refinement and validation.

#### Appendix A.1.3. Post-Processing Steps

**(i) Symmetric normalization** is used to scale the attribution values from a range of [a,b] (where a,b∈Randa<b) to [−1,1].

Given a vector V, the normalization of V such that its components $v_{i}$ are scaled to the interval [−1,1] can be performed using Equation (A2).

(A2) $$v_{i,\mathrm{normalized}}=2\times \frac{\left(v_{i}-V_{\min}\right)}{\left(V_{\max}-V_{\min}\right)}-1$$

where $V_{\min}$ and $V_{\max}$ are the minimum and maximum values of the components of V, respectively.

**(ii) Max-abs normalization** is a non-standard symmetric technique, based on scaling the attribution by the maximum absolute value from the attribution vector to preserve the initial neutral attribution values. This ensures that the values are constrained within the range [−1,1]. This normalization is defined according to Equation (A3):

(A3) $$v_{i,\mathrm{normalized}}=\frac{v_{i}}{\max(|V|)}$$

where |V| denotes the absolute value of attributions.

**(iii) Bipolar range normalization** is a tailored normalization method used to emphasize the effect of each type of attribution (positive, negative, and neutral). A small positive value ϵ is first set to represent the neutral attributions. Positive attributions are scaled to a range of ϵ to 1, while negative attributions are scaled to a range of −1 to −ϵ, with zero values remaining unchanged.

Given a vector $V=(v_{1},v_{2},\ldots ,v_{n})$, each component $v_{i,\mathrm{normalized}}$ of the normalized vector $V_{\mathrm{normalized}}$ is computed according to Equation (A4).

(A4) $$v_{i,\mathrm{normalized}}=\left\{\begin{array}{cc} \frac{v_{i}}{V_{\max}} & \mathrm{if}\;v_{i}>\epsilon ,\\ 0 & \mathrm{if}\;|v_{i}|⩽\epsilon ,\\ \frac{v_{i}}{-V_{\min}} & \mathrm{if}\;v_{i}<-\epsilon . \end{array}\right.$$

where $V_{\min}$ and $V_{\max}$ are the minimum and maximum values of the components of V, respectively.

**Algorithm A2 Clipping step**
**Input:** V (vector of attribution values), *p* (percentile parameter) **Output:** V (post-processed vector) 1: N←length(V) 2: S←sort(V) 3: k←⌈p×N⌉ 4: $V_{\mathrm{high}}\leftarrow S\left[N-k\right]$ 5: $V_{\mathrm{low}}\leftarrow S\left[k-1\right]$ 6: **for** i←1 **to** *N* **do** 7: **if** $V\left[i\right]\geq V_{\mathrm{high}}$ **then** 8: V[i]←S[N−k−1] 9: **else if** $V\left[i\right]\leq V_{\mathrm{low}}$ **then** 10: V[i]←S[k−2] 11: **end if** 12: **end for** 13: **return** V

### Appendix A.4. Explanation Results

#### Appendix A.4.1. Quantitative Results

**Table A1:** Results of the XAI evaluation metrics on the CB55 dataset. Bold values indicate the best values on each metric for each method. The bold values indicate the best recorded metrics for each model on each dataset. The ↑ symbols indicate that higher values are better. The ↓ indicate that the lower values are the better.

L-U-NET|GradCAM								
	Infid↓		Sens_Max↓		CH↑		ACS↑	
**Layer**	**A**	**fA**	**A**	**fA**	**A**	**fA**	**A**	**fA**
*Dec4*	0.86	0.39	0.09	0.06	0.71	0.78	0.57	0.53
*Dec3*	0.85	0.32	0.06	0.05	0.72	0.80	0.74	0.67
*Dec2*	0.73	0.32	0.09	0.04	0.71	0.72	0.74	0.67
*Dec1*	0.72	0.32	0.03	0.03	0.77	0.77	0.64	0.66
*FL*	**0.60**	**0.31**	**0.02**	**0.02**	**0.81**	**0.83**	**0.99**	**0.99**
**S-U-NET|GradCAM**								
	Infid↓		Sens_Max↓		CH↑		ACS↑	
**Layer**	**A**	**fA**	**A**	**fA**	**A**	**fA**	**A**	**fA**
*Dec4*	1.13	0.33	0.06	0.06	0.61	0.65	0.30	0.33
*Dec3*	1.16	0.32	0.02	0.02	0.62	0.60	0.50	0.35
*Dec2*	1.12	**0.31**	0.03	0.03	0.67	0.65	0.73	0.64
*Dec1*	1.17	0.31	0.03	0.03	0.76	0.73	0.75	0.67
*FL*	**1.10**	0.31	**0.01**	**0.01**	**0.80**	**0.83**	**1.00**	**1.00**
**L-U-NET|LRP**								
	Infid↓		Sens_Max↓		CH↑		ACS↑	
**Layer**	**A**	**fA**	**A**	**fA**	**A**	**fA**	**A**	**fA**
*Dec4*	0.85	0.41	1.75	1.89	0.55	0.60	0.48	0.57
*Dec3*	0.85	0.33	1.83	1.98	0.61	0.57	0.63	0.77
*Dec2*	0.83	0.33	1.83	1.86	0.58	0.55	0.73	0.79
*Dec1*	0.71	0.35	1.76	1.85	0.55	0.51	0.73	0.77
*FL*	**0.65**	**0.32**	**0.11**	**0.14**	**0.90**	**0.87**	**0.91**	**0.88**
**Layer**	**A**	**fA**	**A**	**fA**	**A**	**fA**	**A**	**fA**
*Dec4*	1.18	0.33	0.78	0.78	0.49	0.44	0.52	0.45
*Dec3*	1.19	0.35	0.90	0.89	0.54	0.52	0.66	0.66
*Dec2*	1.16	**0.31**	0.97	0.98	0.52	0.48	0.90	0.91
*Dec1*	1.16	0.32	1.31	1.37	0.54	0.50	0.76	0.81
*FL*	**1.10**	0.32	**0.06**	**0.08**	**0.92**	**0.90**	**0.96**	**0.95**
**L-U-NET|DeepLift**								
	Infid↓		Sens_Max↓		CH↑		ACS↑	
**Layer**	**A**	**fA**	**A**	**fA**	**A**	**fA**	**A**	**fA**
*Dec4*	0.87	0.33	0.04	0.02	0.53	0.54	0.77	0.88
*Dec3*	0.84	0.32	**0.03**	**0.02**	0.55	0.55	0.96	0.98
*Dec2*	0.82	0.32	0.04	0.03	0.60	0.61	0.96	0.99
*Dec1*	**0.57**	0.31	0.04	0.04	0.65	0.65	0.98	0.98
*FL*	0.58	**0.31**	0.07	0.08	**0.67**	**0.68**	**0.99**	**0.99**
**S-U-NET|DeepLift**								
	Infid↓		Sens_Max↓		CH↑		ACS↑	
**Layer**	**A**	**fA**	**A**	**fA**	**A**	**fA**	**A**	**fA**
*Dec4*	1.16	0.33	0.05	0.03	0.39	0.41	0.42	0.47
*Dec3*	1.13	0.32	**0.03**	**0.02**	0.60	0.57	0.78	0.94
*Dec2*	1.12	**0.31**	0.04	0.03	0.57	0.54	**0.83**	**1.00**
*Dec1*	**1.08**	0.32	0.05	0.05	0.67	0.71	0.75	0.99
*FL*	1.09	0.32	0.10	0.10	**0.69**	**0.74**	0.76	1.00
**L-U-NET|G*I**								
	Infid↓		Sens_Max↓		CH↑		ACS↑	
**Layer**	**A**	**fA**	**A**	**fA**	**A**	**fA**	**A**	**fA**
*Dec4*	0.82	0.40	0.17	0.14	0.50	0.54	0.65	0.81
*Dec3*	0.80	0.32	**0.08**	**0.05**	0.59	0.54	0.77	**0.98**
*Dec2*	0.78	0.32	0.19	0.08	0.56	0.56	0.95	0.98
*Dec1*	**0.60**	0.32	0.11	0.10	0.56	0.53	**0.96**	0.96
*FL*	0.60	**0.31**	0.13	0.17	**0.63**	**0.62**	0.96	0.94
**S-U-NET|G*I**								
	Infid↓		Sens_Max↓		CH↑		ACS↑	
**Layer**	**A**	**fA**	**A**	**fA**	**A**	**fA**	**A**	**fA**
*Dec4*	1.18	0.33	0.11	0.10	0.55	0.48	0.59	0.53
*Dec3*	1.20	0.35	0.10	0.08	0.57	0.55	0.71	0.70
*Dec2*	1.16	**0.31**	0.05	0.04	0.54	0.53	0.92	0.94
*Dec1*	1.15	0.32	0.04	0.04	0.58	**0.60**	0.98	0.99
*FL*	**1.10**	0.31	**0.02**	**0.03**	**0.58**	0.58	**1.00**	**1.00**

**Table A2:** Results of the XAI evaluation metrics on the UTP-110 dataset. The bold values indicate the best recorded metrics for each model on each dataset. The ↑ symbols indicate that higher values are better. The ↓ indicate that the lower values are the better.

L-U-NET|GradCAM								
	Infid↓		Sens_Max↓		CH↑		ACS↑	
**Layer**	**A**	**fA**	**A**	**fA**	**A**	**fA**	**A**	**fA**
*Dec4*	4.71	1.09	0.10	0.11	0.63	0.61	0.47	0.39
*Dec3*	5.78	1.08	0.07	0.08	0.67	0.67	0.50	0.43
*Dec2*	4.89	1.10	1.70	1.98	0.74	0.73	0.63	0.57
*Dec1*	1.58	1.06	0.04	0.05	0.81	0.79	0.63	0.74
*FL*	**1.29**	**1.03**	**0.04**	**0.04**	**0.86**	**0.85**	**0.96**	**0.95**
**S-U-NET|GradCAM**								
	Infid↓		Sens_Max↓		CH↑		ACS↑	
**Layer**	**A**	**fA**	**A**	**fA**	**A**	**fA**	**A**	**fA**
*Dec4*	3.25	1.41	0.04	0.04	0.64	0.62	0.46	0.47
*Dec3*	2.71	2.50	0.03	0.03	0.65	0.62	0.59	0.55
*Dec2*	3.49	1.21	0.03	0.03	0.72	0.72	0.52	0.60
*Dec1*	3.79	**1.08**	0.02	0.02	0.76	0.78	0.76	0.87
*FL*	**1.42**	1.09	**0.01**	**0.01**	**0.85**	**0.83**	**0.96**	**0.95**
**L-U-NET|LRP**								
	Infid↓		Sens_Max↓		CH↑		ACS↑	
**Layer**	**A**	**fA**	**A**	**fA**	**A**	**fA**	**A**	**fA**
*Dec4*	7.22	2.33	2.18	2.20	0.50	0.48	0.54	0.57
*Dec3*	6.93	2.31	2.21	2.21	0.44	0.42	0.48	0.53
*Dec2*	6.32	2.04	2.40	2.40	0.40	0.38	0.49	0.50
*Dec1*	3.52	2.39	2.51	2.56	0.43	0.40	0.39	0.45
*FL*	**1.31**	**1.05**	**0.15**	**0.16**	**0.94**	**0.93**	**0.94**	**0.93**
**S-U-NET|LRP**								
	Infid↓		Sens_Max↓		CH↑		ACS↑	
**Layer**	**A**	**fA**	**A**	**fA**	**A**	**fA**	**A**	**fA**
*Dec4*	4.94	2.34	1.12	1.10	0.47	0.42	0.59	0.64
*Dec3*	4.51	3.35	1.21	1.07	0.45	0.44	0.63	0.60
*Dec2*	3.77	1.35	1.35	1.26	0.50	0.48	0.66	0.71
*Dec1*	3.95	1.18	1.40	1.33	0.48	0.45	0.57	0.63
*FL*	**1.45**	**1.12**	**0.07**	**0.07**	**0.93**	**0.92**	**0.95**	**0.94**
**L-U-NET|DeepLift**								
	Infid↓		Sens_Max↓		CH↑		ACS↑	
**Layer**	**A**	**fA**	**A**	**fA**	**A**	**fA**	**A**	**fA**
*Dec4*	4.69	1.20	**0.07**	0.06	0.60	0.64	0.82	0.81
*Dec3*	4.98	1.28	0.07	**0.06**	0.63	0.68	0.87	**0.87**
*Dec2*	1.28	1.04	0.09	0.10	0.69	0.69	0.85	0.83
*Dec1*	**1.26**	**1.01**	0.09	0.10	0.73	0.73	**0.89**	0.87
*FL*	1.28	1.03	0.14	0.16	**0.79**	**0.78**	0.88	0.86
**S-U-NET|DeepLift**								
	Infid↓		Sens_Max↓		CH↑		ACS↑	
**Layer**	**A**	**fA**	**A**	**fA**	**A**	**fA**	**A**	**fA**
*Dec4*	1.77	1.28	**0.02**	**0.02**	0.53	0.54	0.80	0.77
*Dec3*	1.88	1.58	0.02	0.02	0.60	0.61	0.91	0.89
*Dec2*	1.52	1.16	0.03	0.03	0.62	0.63	0.92	0.91
*Dec1*	**1.39**	**1.07**	0.03	0.04	0.71	0.71	0.94	0.93
*FL*	1.42	1.10	0.05	0.06	**0.77**	**0.76**	**0.96**	**0.95**
**L-U-NET|G*I**								
	Infid↓		Sens_Max↓		CH↑		ACS↑	
**Layer**	**A**	**fA**	**A**	**fA**	**A**	**fA**	**A**	**fA**
*Dec4*	6.85	1.95	0.33	0.30	0.52	0.49	0.73	0.75
*Dec3*	6.50	1.85	0.22	0.19	0.54	0.52	0.72	0.79
*Dec2*	5.45	1.51	0.27	0.21	0.50	0.48	0.70	0.75
*Dec1*	2.30	1.87	0.28	0.29	0.49	0.47	0.72	0.84
*FL*	**1.29**	**1.05**	**0.07**	**0.08**	**0.75**	**0.74**	**0.96**	**0.95**
**S-U-NET|G*I**								
	Infid↓		Sens_Max↓		CH↑		ACS↑	
**Layer**	**A**	**fA**	**A**	**fA**	**A**	**fA**	**A**	**fA**
*Dec4*	4.82	2.24	0.08	0.07	0.51	0.46	0.67	0.73
*Dec3*	4.10	3.23	0.08	0.07	0.52	0.50	0.83	0.81
*Dec2*	3.55	1.29	0.10	0.08	0.61	0.58	0.86	0.90
*Dec1*	3.83	**1.09**	0.11	0.05	0.68	0.66	0.89	0.93
*FL*	**1.43**	1.11	**0.03**	**0.04**	**0.76**	**0.73**	**0.96**	**0.95**

#### Appendix A.4.3. Assessment

**Table A3:** Performance comparison of GradCAM, LRP, DeepLift, and G*I methods. Each block shows the computed metrics: human assessment (*HA*), pointing game (*PG*), content heatmap (*CH*), and Attribution Concordance Score (*ACS*), across different layers for each XAI method in the CB55 dataset. ✓✓, ✓ and × symbols indicate indicate good, medium and bad evaluation scores receptively.

GradCAM					
**Class**	**Layer**	***HA***	***PG***	***CH***	***AC***
*TXT*	*Dec4*	✓	0.000	0.488	0.495
	*Dec3*	✓✓	1.000	0.613	0.984
	*Dec2*	✓✓	1.000	0.594	0.998
	*Dec1*	✓✓	1.000	0.618	0.999
	*FL*	✓✓	1.000	0.715	0.999
*HL*	*Dec4*	✓	0.308	0.470	0.000
	*Dec3*	✓	0.308	0.584	0.000
	*Dec2*	✓	0.308	0.608	0.019
	*Dec1*	✓✓	0.538	0.620	0.038
	*FL*	✓	0.692	0.757	0.996
*GL*	*Dec4*	✓	0.000	0.645	0.498
	*Dec3*	✓	0.000	0.511	0.153
	*Dec2*	✓	1.000	0.589	0.907
	*Dec1*	✓✓	1.000	0.607	0.982
	*FL*	✓✓	1.000	0.712	0.993
**LRP**					
**Class**	**Layer**	***HA***	***PG***	***CH***	***AC***
*TXT*	*Dec4*	✓✓	0.100	0.534	0.518
	*Dec3*	✓✓	0.850	0.391	0.929
	*Dec2*	✓	0.700	0.419	0.922
	*Dec1*	✓	0.700	0.526	0.855
	*FL*	✓✓	0.500	0.923	0.966
*HL*	*Dec4*	✓✓	0.462	0.408	0.200
	*Dec3*	✓✓	0.615	0.532	0.818
	*Dec2*	✓✓	0.692	0.456	0.853
	*Dec1*	✓✓	0.538	0.335	0.724
	*FL*	✓✓	0.308	0.890	0.953
*GL*	*Dec4*	✓✓	0.278	0.440	0.597
	*Dec3*	✓✓	0.222	0.555	0.303
	*Dec2*	✓✓	0.833	0.496	0.899
	*Dec1*	✓✓	0.667	0.553	0.799
	*FL*	✓✓	0.333	0.872	0.943
**DeepLift**					
**Class**	**Layer**	***HA***	***PG***	***CH***	***AC***
*TXT*	*Dec4*	✓✓	0.000	0.367	0.573
	*Dec3*	✓✓	1.000	0.523	0.995
	*Dec2*	×	1.000	0.543	0.999
	*Dec1*	×	1.000	0.712	0.995
	*FL*	×	1.000	0.726	0.999
*HL*	*Dec4*	✓✓	0.385	0.398	0.205
	*Dec3*	✓✓	0.692	0.571	0.988
	*Dec2*	✓✓	0.692	0.571	0.999
	*Dec1*	✓✓	0.692	0.755	0.991
	*FL*	✓✓	0.692	0.789	0.994
*GL*	*Dec4*	✓	0.000	0.505	0.618
	*Dec3*	×	0.333	0.568	0.850
	*Dec2*	×	1.000	0.487	0.993
	*Dec1*	×	1.000	0.665	0.988
	*FL*	×	1.000	0.700	0.996
**G*I**					
**Class**	**Layer**	***HA***	***PG***	***CH***	***AC***
*TXT*	*Dec4*	✓✓	0.000	0.493	0.627
	*Dec3*	✓✓	0.950	0.497	0.994
	*Dec2*	✓✓	1.000	0.523	0.999
	*Dec1*	✓✓	1.000	0.585	0.998
	*FL*	✓✓	1.000	0.574	0.999
*HL*	*Dec4*	✓✓	0.462	0.486	0.314
	*Dec3*	✓✓	0.692	0.539	0.777
	*Dec2*	✓✓	0.692	0.511	0.843
	*Dec1*	✓✓	0.692	0.592	0.973
	*FL*	✓✓	0.692	0.564	0.996
*GL*	*Dec4*	✓✓	0.278	0.500	0.636
	*Dec3*	✓✓	0.111	0.527	0.399
	*Dec2*	✓✓	1.000	0.524	0.987
	*Dec1*	✓✓	1.000	0.607	0.991
	*FL*	✓✓	1.000	0.575	0.996

**Table A4:** Performance comparison of GradCAM, LRP, DeepLift, and G*I methods. Each panel shows the computed metrics: human assessment (*HA*), pointing game (*PG*), content heatmap (*CH*), and Attribution Concordance Score (*AC*), across layers on UTP-110. ✓✓, ✓ and × symbols indicate indicate good, medium and bad evaluation scores receptively.

GradCAM					
**Class**	**Layer**	***HA***	***PG***	***CH***	***AC***
*DEC*	*Dec4*	✓	0.737	0.610	0.938
	*Dec3*	✓✓	0.947	0.670	0.991
	*Dec2*	✓✓	0.947	0.742	1.000
	*Dec1*	✓✓	0.947	0.761	0.999
	*FL*	✓✓	0.947	0.870	0.999
*FIL*	*Dec4*	×	0.059	0.672	0.166
	*Dec3*	×	0.000	0.596	0.149
	*Dec2*	✓	0.882	0.633	0.261
	*Dec1*	✓	1.000	0.681	0.785
	*FL*	✓✓	1.000	0.814	0.998
*TXTL*	*Dec4*	✓	0.650	0.670	0.817
	*Dec3*	✓✓	1.000	0.509	0.967
	*Dec2*	✓✓	1.000	0.696	0.997
	*Dec1*	✓	1.000	0.709	0.984
	*FL*	✓✓	1.000	0.797	0.999
*BD*	*Dec4*	✓	0.950	0.583	0.686
	*Dec3*	✓✓	0.800	0.623	0.928
	*Dec2*	✓✓	1.000	0.671	0.949
	*Dec1*	✓✓	1.000	0.746	0.983
	*FL*	✓✓	1.000	0.820	0.986
*SInit*	*Dec4*	×	0.632	0.584	0.150
	*Dec3*	×	0.158	0.550	0.078
	*Dec2*	✓	0.842	0.593	0.175
	*Dec1*	✓	0.895	0.693	0.903
	*FL*	✓✓	0.895	0.809	0.997
*BInit*	*Dec4*	×	0.632	0.476	0.159
	*Dec3*	✓	0.526	0.622	0.255
	*Dec2*	✓	0.895	0.623	0.535
	*Dec1*	✓✓	0.895	0.672	0.947
	*FL*	✓✓	0.895	0.775	0.995
**LRP**					
**Class**	**Layer**	***HA***	***PG***	***CH***	***AC***
*DEC*	*Dec4*	✓	0.579	0.777	0.586
	*Dec3*	✓✓	0.474	0.467	0.468
	*Dec2*	✓✓	0.526	0.511	0.575
	*Dec1*	✓	0.474	0.458	0.504
	*FL*	✓✓	0.368	0.994	0.996
*FIL*	*Dec4*	✓✓	0.882	0.415	0.802
	*Dec3*	✓✓	0.882	0.317	0.851
	*Dec2*	✓✓	0.941	0.452	0.917
	*Dec1*	✓✓	0.824	0.534	0.919
	*FL*	✓✓	0.353	0.961	0.975
*TXTL*	*Dec4*	✓✓	0.700	0.349	0.915
	*Dec3*	✓✓	0.950	0.371	0.892
	*Dec2*	✓✓	0.750	0.545	0.995
	*Dec1*	✓✓	0.450	0.346	0.993
	*FL*	✓✓	0.300	0.985	0.987
*BD*	*Dec4*	✓	0.550	0.303	0.623
	*Dec3*	✓	0.700	0.542	0.510
	*Dec2*	✓✓	0.300	0.641	0.772
	*Dec1*	✓✓	0.400	0.476	0.435
	*FL*	✓✓	0.050	0.952	0.967
*SInit*	*Dec4*	✓✓	0.789	0.350	0.620
	*Dec3*	✓✓	0.579	0.630	0.563
	*Dec2*	✓✓	0.632	0.447	0.812
	*Dec1*	✓✓	0.632	0.364	0.737
	*FL*	✓✓	0.526	0.986	0.980
*BInit*	*Dec4*	✓✓	0.684	0.498	0.647
	*Dec3*	✓✓	0.579	0.507	0.653
	*Dec2*	✓✓	0.474	0.524	0.610
	*Dec1*	✓✓	0.579	0.514	0.542
	*FL*	✓✓	0.316	0.883	0.978
**DeepLift**					
**Class**	**Layer**	***HA***	***PG***	***CH***	***AC***
*DEC*	*Dec4*	✓	0.842	0.797	0.906
	*Dec3*	✓✓	0.789	0.734	0.884
	*Dec2*	✓✓	0.895	0.600	0.993
	*Dec1*	✓✓	0.947	0.640	0.995
	*FL*	✓✓	0.947	0.748	0.996
*FIL*	*Dec4*	✓✓	1.000	0.620	0.946
	*Dec3*	✓✓	1.000	0.576	1.000
	*Dec2*	✓✓	1.000	0.720	0.997
	*Dec1*	✓✓	1.000	0.752	0.997
	*FL*	✓✓	1.000	0.836	0.999
*TXTL*	*Dec4*	✓✓	1.000	0.670	0.913
	*Dec3*	✓✓	1.000	0.477	0.999
	*Dec2*	✓✓	1.000	0.651	0.998
	*Dec1*	✓✓	1.000	0.761	0.999
	*FL*	✓✓	1.000	0.814	1.000
*BD*	*Dec4*	✓	0.850	0.373	0.598
	*Dec3*	✓✓	0.850	0.664	0.918
	*Dec2*	✓✓	1.000	0.651	0.981
	*Dec1*	✓✓	1.000	0.767	0.984
	*FL*	✓✓	1.000	0.802	0.990
*SInit*	*Dec4*	✓✓	0.895	0.464	0.996
	*Dec3*	✓✓	0.895	0.677	0.997
	*Dec2*	✓✓	0.895	0.755	0.996
	*Dec1*	✓✓	0.895	0.788	0.995
	*FL*	✓✓	0.895	0.833	0.997
*BInit*	*Dec4*	✓✓	0.895	0.481	0.995
	*Dec3*	✓✓	0.895	0.664	0.999
	*Dec2*	✓✓	0.895	0.692	0.997
	*Dec1*	✓✓	0.895	0.727	0.997
	*FL*	✓✓	0.895	0.747	0.999
**G*I**					
**Class**	**Layer**	***HA***	***PG***	***CH***	***AC***
*DEC*	*Dec4*	✓	0.737	0.823	0.821
	*Dec3*	✓✓	0.579	0.458	0.891
	*Dec2*	✓✓	0.789	0.577	0.983
	*Dec1*	✓✓	0.842	0.604	0.999
	*FL*	✓✓	0.947	0.833	0.999
*FIL*	*Dec4*	✓✓	1.000	0.477	0.936
	*Dec3*	✓✓	1.000	0.391	0.955
	*Dec2*	✓✓	1.000	0.622	0.995
	*Dec1*	✓✓	1.000	0.724	0.998
	*FL*	✓✓	1.000	0.792	0.999
*TXTL*	*Dec4*	✓✓	0.800	0.511	0.981
	*Dec3*	✓✓	1.000	0.437	0.907
	*Dec2*	✓✓	1.000	0.573	0.998
	*Dec1*	✓✓	1.000	0.747	0.997
	*FL*	✓✓	1.000	0.758	1.000
*BD*	*Dec4*	✓	0.600	0.283	0.602
	*Dec3*	✓✓	0.900	0.632	0.673
	*Dec2*	✓✓	1.000	0.694	0.995
	*Dec1*	✓✓	1.000	0.719	0.991
	*FL*	✓✓	1.000	0.744	0.991
*SInit*	*Dec4*	✓✓	0.789	0.402	0.685
	*Dec3*	✓✓	0.789	0.731	0.866
	*Dec2*	✓✓	0.842	0.605	0.991
	*Dec1*	✓✓	0.737	0.730	0.992
	*FL*	✓✓	0.895	0.770	0.998
*BInit*	*Dec4*	✓✓	0.737	0.485	0.740
	*Dec3*	✓✓	0.789	0.652	0.917
	*Dec2*	✓✓	0.684	0.556	0.919
	*Dec1*	✓✓	0.895	0.634	0.988
	*FL*	✓✓	0.895	0.685	0.999

### Appendix A.6. Generalization Section

**Table A5:** Comparison of attribution methods: GradCAM, LRP, DeepLift, and G*I. Each block shows the computed metrics—human assessment (*HA*), pointing game (*PG*), content heatmap (*CH*), and Attribution Concordance Score (*ACS*)—across layers for each XAI method on the CityScapes dataset. ✓✓, ✓ and × symbols indicate good, medium and bad evaluation scores receptively.

GradCAM					
**Class**	**Layer**	***HA***	***PG***	***CH***	***AC***
road	*Dec4*	×	0.905	0.585	0.933
	*Dec3*	×	0.905	0.725	0.944
	*Dec2*	✓✓	0.905	0.700	0.943
	*Dec1*	✓✓	0.905	0.740	0.947
	*FL*	✓✓	0.905	0.801	0.945
sidewalk	*Dec4*	×	0.609	0.560	0.668
	*Dec3*	×	0.739	0.643	0.756
	*Dec2*	✓	0.783	0.650	0.816
	*Dec1*	✓	0.696	0.685	0.802
	*FL*	✓	0.696	0.697	0.800
person	*Dec4*	×	0.261	0.542	0.045
	*Dec3*	×	0.435	0.505	0.191
	*Dec2*	×	0.261	0.549	0.080
	*Dec1*	×	0.304	0.482	0.176
	*FL*	✓	0.609	0.576	0.562
car	*Dec4*	×	0.850	0.546	0.682
	*Dec3*	×	0.850	0.535	0.709
	*Dec2*	✓	0.900	0.594	0.724
	*Dec1*	✓	0.900	0.573	0.854
	*FL*	✓✓	0.950	0.626	0.906
bus	*Dec4*	×	0.500	0.576	0.500
	*Dec3*	×	0.667	0.529	0.959
	*Dec2*	×	0.667	0.627	0.910
	*Dec1*	×	0.500	0.654	0.761
	*FL*	✓	0.667	0.684	0.999
**LRP**					
**Class**	**Layer**	***HA***	***PG***	***CH***	***AC***
road	*Dec4*	×	0.857	0.491	0.935
	*Dec3*	×	0.905	0.621	0.944
	*Dec2*	✓✓	0.810	0.626	0.943
	*Dec1*	✓✓	0.762	0.782	0.946
	*FL*	✓✓	0.333	0.971	0.937
sidewalk	*Dec4*	×	0.696	0.392	0.792
	*Dec3*	×	0.783	0.481	0.848
	*Dec2*	✓	0.391	0.443	0.855
	*Dec1*	✓	0.391	0.479	0.862
	*FL*	✓	0.217	0.749	0.831
person	*Dec4*	×	0.435	0.509	0.359
	*Dec3*	×	0.609	0.410	0.463
	*Dec2*	✓	0.522	0.379	0.597
	*Dec1*	✓	0.348	0.358	0.624
	*FL*	✓	0.261	0.571	0.601
car	*Dec4*	×	0.700	0.403	0.798
	*Dec3*	×	0.700	0.418	0.815
	*Dec2*	✓	0.350	0.415	0.782
	*Dec1*	✓	0.250	0.407	0.766
	*FL*	✓✓	0.300	0.863	0.843
bus	*Dec4*	×	0.667	0.414	0.933
	*Dec3*	×	0.667	0.308	0.997
	*Dec2*	✓	0.500	0.356	0.992
	*Dec1*	✓	0.667	0.218	0.942
	*FL*	✓	0.333	0.631	0.985
**DeepLift**					
**Class**	**Layer**	***HA***	***PG***	***CH***	***AC***
road	*Dec4*	×	0.905	0.340	0.893
	*Dec3*	×	0.905	0.361	0.902
	*Dec2*	✓	0.905	0.374	0.927
	*Dec1*	✓	0.810	0.431	0.928
	*FL*	✓✓	0.810	0.472	0.931
sidewalk	*Dec4*	×	0.783	0.329	0.809
	*Dec3*	×	0.783	0.404	0.860
	*Dec2*	✓	0.783	0.406	0.880
	*Dec1*	✓	0.739	0.457	0.885
	*FL*	✓	0.783	0.468	0.879
person	*Dec4*	✓	0.348	0.463	0.321
	*Dec3*	×	0.565	0.349	0.563
	*Dec2*	✓	0.609	0.297	0.647
	*Dec1*	✓	0.565	0.343	0.648
	*FL*	✓	0.565	0.389	0.618
car	*Dec4*	×	0.850	0.357	0.839
	*Dec3*	×	0.900	0.391	0.909
	*Dec2*	✓✓	0.950	0.495	0.927
	*Dec1*	✓✓	0.950	0.563	0.922
	*FL*	✓✓	0.950	0.593	0.925
bus	*Dec4*	×	0.667	0.394	0.980
	*Dec3*	×	0.667	0.318	0.998
	*Dec2*	✓	0.667	0.427	0.998
	*Dec1*	✓	0.667	0.473	0.996
	*FL*	✓	0.667	0.494	0.998
**G*I**					
**Class**	**Layer**	***HA***	***PG***	***CH***	***AC***
road	*Dec4*	×	0.905	0.498	0.938
	*Dec3*	×	0.905	0.604	0.944
	*Dec2*	✓	0.905	0.609	0.944
	*Dec1*	✓✓	0.905	0.660	0.946
	*FL*	✓✓	0.905	0.775	0.947
sidewalk	*Dec4*	×	0.696	0.364	0.799
	*Dec3*	×	0.783	0.446	0.865
	*Dec2*	✓	0.739	0.420	0.886
	*Dec1*	✓	0.739	0.477	0.890
	*FL*	✓	0.696	0.521	0.878
person	*Dec4*	×	0.391	0.502	0.357
	*Dec3*	×	0.609	0.401	0.477
	*Dec2*	✓	0.522	0.352	0.605
	*Dec1*	✓	0.565	0.352	0.652
	*FL*	✓	0.609	0.333	0.646
car	*Dec4*	×	0.900	0.371	0.829
	*Dec3*	×	0.900	0.409	0.904
	*Dec2*	✓✓	0.850	0.486	0.921
	*Dec1*	✓✓	0.850	0.505	0.921
	*FL*	✓✓	0.950	0.559	0.933
bus	*Dec4*	×	0.667	0.414	0.920
	*Dec3*	×	0.667	0.278	0.954
	*Dec2*	✓	0.667	0.357	0.998
	*Dec1*	✓	0.667	0.456	0.992
	*FL*	✓	0.667	0.436	0.998
